# Mechanistic insights into the early life stage microbiota of silver pompano (*Trachinotus blochii*)

**DOI:** 10.3389/fmicb.2024.1356828

**Published:** 2024-04-17

**Authors:** T. G. Sumithra, S. R. Krupesha Sharma, Gayathri Suresh, Ambarish P. Gop, S. Surya, P. Gomathi, M. K. Anil, K. A. Sajina, K. J. Reshma, Sanal Ebeneezar, Iyyapparaja Narasimapallavan, A. Gopalakrishnan

**Affiliations:** ^1^Marine Biotechnology, Fish Nutrition, and Health Division, ICAR-Central Marine Fisheries Research Institute (CMFRI), Kochi, India; ^2^Cochin University of Science and Technology, Kochi, Kerala, India; ^3^Vizhinjam Regional Centre of ICAR-Central Marine Fisheries Research Institute, Thiruvananthapuram, Kerala, India

**Keywords:** marine aquaculture, larval microbiota, gut microbiota, host–microbe interactions, egg microbiota

## Abstract

**Introduction:**

Deep investigations of host-associated microbiota can illuminate microbe-based solutions to improve production in an unprecedented manner. The poor larval survival represents the critical bottleneck in sustainable marine aquaculture practices. However, little is known about the microbiota profiles and their governing eco-evolutionary processes of the early life stages of marine teleost, impeding the development of suitable beneficial microbial management strategies. The study provides first-hand mechanistic insights into microbiota and its governing eco-evolutionary processes in early life stages of a tropical marine teleost model, *Trachinotus blochii*.

**Methods:**

The microbiota profiles and their dynamics from the first day of hatching till the end of metamorphosis and that of fingerling’s gut during the routine hatchery production were studied using 16S rRNA amplicon-based high-throughput sequencing. Further, the relative contributions of various external factors (rearing water, live feed, microalgae, and formulated feed) to the microbiota profiles at different ontogenies was also analyzed.

**Results:**

A less diverse but abundant core microbial community (~58% and 54% in the whole microbiota and gut microbiota, respectively) was observed throughout the early life stages, supporting ‘core microbiota’ hypothesis. Surprisingly, there were two well-differentiated clusters in the whole microbiota profiles, ≤10 DPH (days post-hatching) and > 10 DPH samples. The levels of microbial taxonomic signatures of stress indicated increased stress in the early stages, a possible explanation for increased mortality during early life stages. Further, the results suggested an adaptive mechanism for establishing beneficial strains along the ontogenetic progression. Moreover, the highly transient microbiota in the early life stages became stable along the ontogenetic progression, hypothesizing that the earlier life stages will be the best window to influence the microbiota. The egg microbiota also crucially affected the microbial community. Noteworthily, both water and the feed microbiota significantly contributed to the early microbiota, with the feed microbiota having a more significant contribution to fish microbiota. The results illustrated that rotifer enrichment would be the optimal medium for the early larval microbiota manipulations.

**Conclusion:**

The present study highlighted the crucial foundations for the microbial ecology of *T. blochii* during early life stages with implications to develop suitable beneficial microbial management strategies for sustainable mariculture production.

## Highlights

- Microbial taxonomic signatures of stress were higher in earlier life stages.
- Microbiota profiles supported the “core microbiota” hypothesis.
- Feed microbiota contributed more to fish microbiota than water microbiota.
- Egg microbiota showed significant similarity with microbiota of *T. blochii*.

## Introduction

The driving force for unraveling the microbiota as an emerging paradigm in biological research is the reassurance that symbiotic microbial communities are critical for ensuring the well-being of their eukaryotic hosts (Luan et al., 2023). The promising data from the microbiota research reveals that the host–microbe interactions co-regulate fundamental aspects of host’s physiology through various mechanisms (Luan et al., 2023). As a result, microbiota-based management strategies are becoming sources of novel and environmentally sustainable alternatives for improving host and ecosystem health during sustainable production practices (Borges et al., 2021). Among other vertebrates, the uniqueness of teleost due to complex lifecycles with metamorphosis and multiple ontogenetic stages, and high dependence on ecotypes complicates the research on teleost microbiota. Although fish are the most diverse and widely distributed vertebrates, only scattered data is available on their microbial ecology and the influencing factors on microbial communities’ acquisition, dynamics, and structuring (Minich et al., 2021). The composite dataset in this context will benefit fish biologists interested in microbial ecology and researchers studying vertebrate microbiota, with profound implications for aquaculture and fishery management (Minich et al., 2021).

Aquaculture forms the world’s fastest-growing food production sector and the most promising option for achieving food and nutrition security. Although technologies for marine fish larviculture have been persistently improved, poor larval survival represents a significant bottleneck in any hatchery practices (Anil et al., 2021). With growing evidence and appeal, sustainable microbial management approaches can potentially enhance larval quality and survival (Borges et al., 2021). However, only scarce data is available on the larval microbiota profiles of maricultured teleosts, while the information is indispensable in generating beneficial larval microbiota manipulation strategies. Identifying the factors and processes governing the colonization of the larval microbiota is also critical in this context (Llewellyn et al., 2014). Deep investigations of host-associated microbiota through high-throughput sequencing technologies can illuminate microbiota dynamics and their influential factors, and aid the engineering of microbiota-based solutions to disease prevention in an unprecedented manner (Borges et al., 2021). Even though deep sequencing data based on 16S rDNA amplicons of teleost microbiota are now accumulating, the data on the whole microbiota of the early life stages are limited (Duval et al., 2022; Lokesh et al., 2024). Further, the transition during the ontogeny of fish and the establishment of their microbial communities are relatively less explored. Limited studies on the microbiota of larval *Gadus morhua* and *Kryptolebias marmoratus* (Bakke et al., 2015; Forberg et al., 2016) and the gut microbiota during the ontogeny of *Danio rerio* (Wong et al., 2015; Stephens et al., 2016), *Ictalurus punctatus* (Bledsoe et al., 2016), *Symphysodon aequifasciata* (Sylvain and Derome, 2017), *Silurus meridionalis* (Zhang et al., 2018), *Salmo salar* (Lokesh et al., 2019), *Seriola lalandi* (Minich et al., 2021) and *Procambarus clarkii* (Xie et al., 2021) have shed particular lights on the importance of early life stage-microbiota for adult fish health. The results suggested that microbial colonization throughout the development of the fish is a function of both exposure and host selection (Borges et al., 2021; Minich et al., 2021). The best chance of successfully managing the complex hatchery systems lies in understanding and controlling the microbial community in each of the different compartments: fish larvae, rearing water, microalgae, and live food (Vadstein et al., 2018). However, no studies have been conducted to investigate these factors throughout the larval rearing period, even though the data is of fundamental interest from both a scientific and commercial lens (Bakke et al., 2015).

In this context, the present paper provides first-hand insights into the early ontogenetic stages of one high-value tropical marine teleost species *Trachinotus blochii* (silver pompano). The microbiota profiles and their dynamics from the first day of hatching till the end of metamorphosis and that of fingerling’s gut during the routine hatchery production were studied using 16S rRNA amplicon-based high-throughput sequencing. The core microbiota, which denotes the persistent microbial members appearing in several assemblages of a particular host ontogeny is a popular term in the research focusing on host–microbe interactions (Walburn et al., 2019). Hence, the core microbiota and the prospective stage-specific microbial signatures were defined from the present results to index larval and fingerling’s health. Further, an in-depth analysis of the relative contributions of various external factors (rearing water, live feed, microalgae, and formulated feed) to the microbiota profiles at different ontogenies was done to characterize the dynamic relationships between the host microbiota, ontogeny, and the environmental microbiota. The results provide several novel insights into the microbiota of *T. blochii* during early life stages with final implications to develop suitable beneficial microbial management strategies within a marine hatchery system.

## Materials and methods

### Experimental animals and hatchery management

The animals were raised in the national brood bank facility of silver pompano at the Vizhinjam Regional Centre of ICAR-Central Marine Fisheries Research Institute, India. The larvae were reared according to standard production practices (Anil et al., 2021). The experiments were done as per the ARRIVE strategies (Percie du Sert et al., 2020). The handling of live animals was done following the guidelines in UK Animals (Scientific Procedures) Act (1986) and EU Directive 2010/63/EU for animal experimentation (2019). ICAR-CMFRI, Kochi, India, permitted the protocols for handling live animals (BT/AAQ/3/SP28267/2018).

### Sampling

We collected different early ontogenetic stages of *T. blochii* from the newly hatched larvae till the completion of metamorphosis, and gut from fingerlings successively from a batch representing a consistent normal cycle. Each sampling point represented an ontogenetic stage maintained on varying feeds. In detail, fertilized eggs, newly hatched larvae/ endogenous feeding stage (1-day post-hatching, DPH), larvae at the beginning of exogenous feeding (3 DPH), larvae fed with rotifers (5 and 7 DPH), larvae fed with rotifer and artemia (10 and 12 DPH), larvae on formulated feeds (20 and 25 DPH) and gut of fingerlings (31 and 75 DPH) were collected. Whole organisms were used for early ontogenetic stages, whereas only intestine was sampled for fingerlings. Approximately 0.1 g (wet weight) larvae pooled (Supplementary Table 1) randomly from one tank constituted one replicate. Likewise, larvae from three tanks were sampled to get triplicates for each stage. The larval pools were transferred to 0.5 mL absolute ethanol (HiMedia, India) and frozen at −20°C (Song et al., 2016; Li et al., 2023). For the fingerlings, fish from three tanks were sampled with three fish from each tank. The pooled gut samples from three fish in the same tank constituted a replicate. The gut with the contents was transferred to 0.5 mL absolute ethanol, and preserved at −20°C (Song et al., 2016; Li et al., 2023). Accordingly, there were 33 fish samples (11 life stages in triplicates) for sequencing. Additionally, 1 L of tank water from each tank at different sampling points (corresponding to 11 life stages) was filtered initially onto a 0.45 μm and then onto 0.22 μm nitrocellulose membrane filters. The 0.22 μm membrane was transferred to 3 mL absolute ethanol and frozen at −20°C (33 samples for sequencing). The microalgae (*Isochrysis galbana* + *Nannochloropsis salina*; 3 samples) and corresponding feed in each stage were also sampled. In detail, the feed collected included rotifers (*Brachionus plicatilis* + *Brachionus rotundiformis*) collected on 1, 3, 5, and 7 DPH (12 samples), *Artemia nauplii* collected on 10 and 12 DPH (6 samples), and different formulated feeds [pellet size of 150 to 200 μM (E-larval 200, Lucky star), 500 μM, 1 mM, 2.5 mM, and 4 mM (Skretting)] (15 feed samples corresponding to five different pellet sizes in triplicates). Around 0.5 g of each feed were collected in 3 mL absolute ethanol at random in triplicates and preserved at −20°C (Song et al., 2016; Li et al., 2023). In detail, the sample numbers for fish, water, microalgae, rotifers, artemia, and formulated feed samples for sequencing was 33, 33, 3, 12, 6, and 15, respectively.

### DNA extraction

There was a total of 102 samples for DNA extraction and sequencing. The samples were initially homogenized in phosphate-buffered saline (PBS; HiMedia, India) with a mortar and pestle, and the homogenate was processed to reduce the host cell contamination as described earlier (Sumithra et al., 2022b). Finally, the metagenomic DNA was extracted from the preparations representing weakly and firmly connected bacteria in the tissue using QIAamp DNA Microbiome Kit (QIAGEN Inc., Toronto, ON, Canada) as per manufacturer’s protocol.^1^ The DNA concentrations were determined using a Qubit Fluorimeter (V.3.0) and preserved at −20°C.

### Preparation of 2 × 300 MiSeq library, cluster generation, and sequencing

The amplicon library was prepared using Nextera XT Index Kit (Illumina Inc.) as per the 16S metagenomic sequencing library preparation protocol (Part # 155044223 Rev. B.) at a custom sequencing facility (Eurofins Genomics Lab, India). The primers 16S rRNA F—5′-GCCTACGGGNGGCWGCAG-3′ and 16S rRNA R—5′-ACTACHVGGGTATCTAATCC-3′ were used for the amplification of the V3-V4 region of 16S rRNA genes. The amplified product was visualized on 1.2% agarose gel. Quality control included checking the size and purity (nonspecific amplicons, if any) of the amplicons in the gel. The quality control (QC) passed amplicons with the Illumina adaptor were amplified using i5 and i7 primers to add multiplexing index sequences and common adapters required for cluster generation as per the standard Illumina protocol. The amplicon libraries were purified by AMPure XP beads, quantified using Qubit Fluorometer, and analyzed on a 4200 Tape Station system (Agilent Technologies) using D1000 Screen tape as per manufacturer instructions. After obtaining the mean peak size (595 to 610 bp) from the Tape station profile, each sample library was normalized to 4 nM concentration, and the normalized libraries were pooled. The 12 pM libraries spiked with 15% PhiX control were loaded into the Illumina MiSeq platform (600 cycles) for cluster generation and paired-end sequencing (2 × 300 bp paired-end mode).

### Bioinformatics analysis

The raw reads generated through the Illumina sequencing platform were checked for quality using the FastQC tool (version 0.11.8) with default parameters after demultiplexing. The base quality (“Phred quality score” > 30 was used as the cut off value), GC content, base composition, adapter dimers, and ambiguous bases (apart from A, T, G, and C) were thoroughly investigated. The forward and reverse primer sequences were maintained to retain all the possible sequence data on the 16SrRNA gene. The reads were then analyzed using the Quantitative Insights into Microbial Ecology pipeline (QIIME2™ version 2023.2.0; Bolyen et al., 2019). Initially, demultiplexed pair-end reads were merged, filtered, and denoised using the Divisive Amplicon Denoising Algorithm 2 (DADA2) to obtain the feature table and representative sequences (Callahan et al., 2016). The sequence data across the samples were not rarefied to avoid losing important information as per previous recommendations (McMurdie and Holmes, 2014; Calle, 2019). The Nave Bayesian classifier was subsequently used to assign taxonomic information to the acquired amplicon sequence variations (ASVs) against the SILVA database version 138, where ASVs were clustered by 99% homology. The ANCOM plugin was then used to calculate the relative abundance of each taxonomic level within the samples. The diversity measures were calculated in QIIME2 using the core metrics pipeline. The PICRUSt2 tool was used to predict metagenome functions such as KEGG orthologs and pathways (Douglas et al., 2020).

### Statistical analysis

The Shapiro–Wilk and Levene tests, respectively were used to determine the normality and homogeneity of variance in the data related to α-diversity measures. The one-way ANOVA followed by *post hoc* analysis through Tukey’s HSD test was used to compare the “normal” data across different studied groups. The Kruskal-Wallis H test was applied to the data that lacked normality. The SPSS (version 16) was used for the analysis. Principal Coordinate Analysis (PCoA) based on the Bray–Curtis similarity index of the ASV abundance profiles was performed to compare the microbiota data between different groups using PAST 3.26 software (Hammer et al., 2001). Further, PCoA based on the weighted UniFrac distance of the ASV abundance profiles was also calculated using the QIIME2 diversity core-metrics-phylogenetic command. The statistical significance of the clustering pattern revealed through the PCoA based on both β-metrics was checked using one-way permutational multivariate analysis of variance (PERMANOVA), and *F*-values and *p*-values were noted. The relative abundance of ASV of the taxa accounting for the top 20 members was used to generate the relative abundance plot in each studied group. The abundance data for the genus were transformed into presence (1) and absence (0) data to identify the core microbiota. The sum of each unique genera belonging to the required group was divided by the total number of samples in that group to estimate the sample coverage in %. The core microbiota members were selected from the genera in >50% of the samples (Borroni et al., 2022; Diez-Méndez et al., 2023). The changes in the relative abundance% of the core microbes along with the growth of silver pompano (DPH wise) were also calculated. Further, Venn diagram analysis identifying the shared core microbes between the different clusters was performed in R software (Version: R-4.3.0) using the “VennDiagram” package. Linear discriminant analysis (LDA) effect size (LEfSe, Galaxy v1.0) was used to identify significantly different relative abundances of bacterial taxa associated with the identified clusters. The threshold value on the logarithmic LDA score for discriminative features was set at 2.0, and the settings for LEfSe analyses were set at α-value of 0.05 for the factorial Kruskal-Walli test among different clusters. The “all against all strategy” was selected for multiclass analysis (Segata et al., 2011). The horizontal bar plots were used to visualize the identified biomarkers by LEfSe ranking. Further, the PICRUSt results on KEGG pathways were analyzed through LEfSe analysis. The Pearson’s correlations representing the correlation between the abundances of ASV features of the identified ontogenetic clusters with their corresponding possible microbiota resources (feed and water) were calculated, and a matrix plot representing the strength and significance of correlations was designed using PAST 3.26 software (Hammer et al., 2001). The relative contributions of different feed and water on each identified ontogenetic cluster were analyzed through one-way PERMANOVA and the *P*- and *F*-values in the pairwise comparison. In all the tests, the differences were interpreted as significant at *p*-values ≤0.05. To estimate the contributions of ASV features from each of the studied sources (microalgae, rotifer, artemia, formulated feed, and water) to the sink communities (whole microbiota profiles of the identified ontogenetic clusters and fingerling gut microbiota), the popular microbial source tracking package “Feast” was performed using R-software (Version: R-4.3.0). The results in the “Feast” were plotted using a 2D pie chart through an online platform for data visualization.^2^

## Results

### Whole microbiota profiles of *Trachinotus blochii* larvae

**Figure 1:**
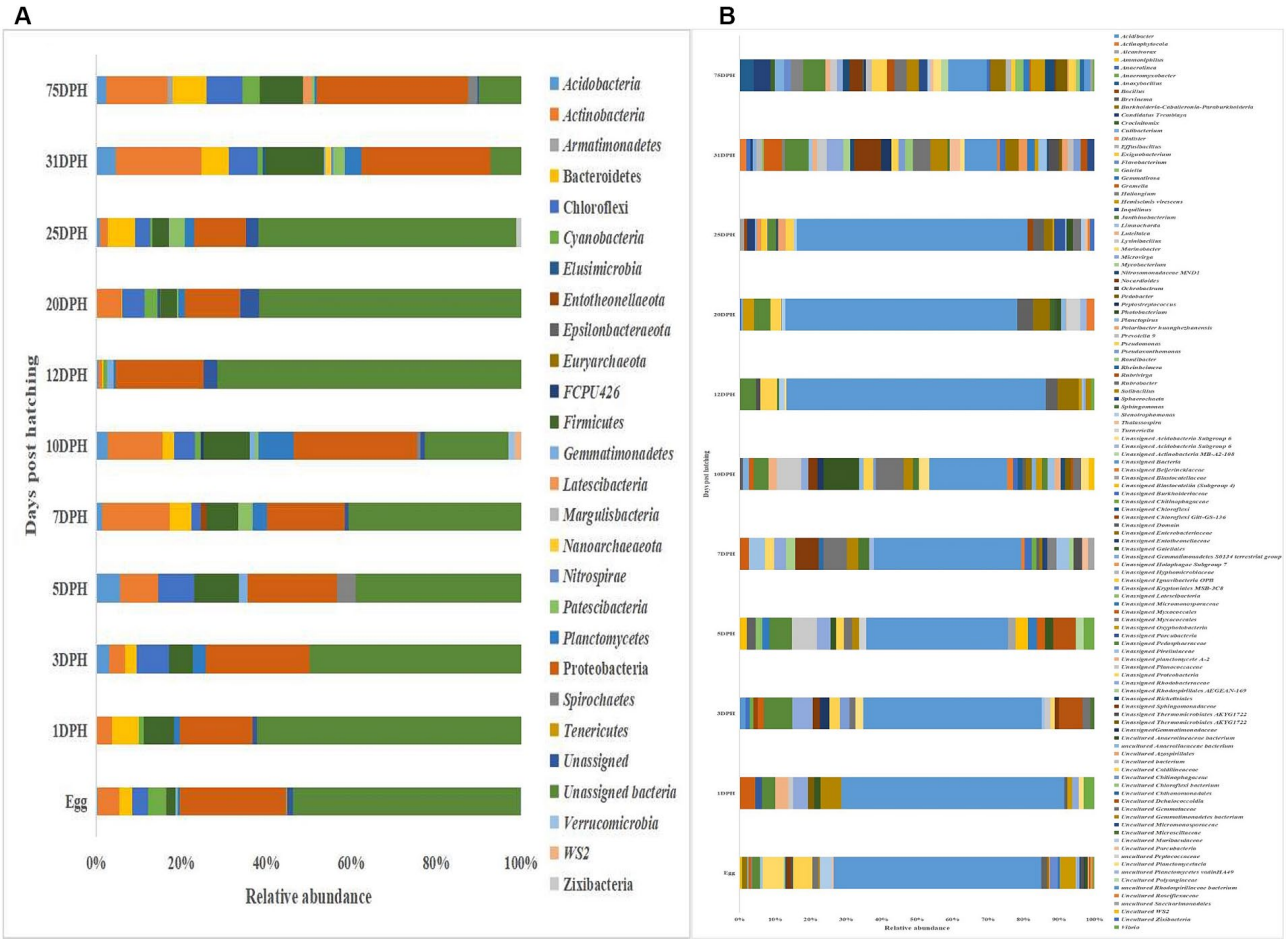
Taxonomic landscape in the microbiota profiles across different ontogenetic stages of *Trachinotus blochii*. **(A)** At phylum level; **(B)** At genus level Taxa representing >0.1% of total abundance were only considered for making plots. For genus-level data, only the top 50 genera were used for plotting. DPH, Days post-hatching.

The metagenomic data sets generated were deposited in the NCBI Sequence Read Archive under Bioproject PRJNA765138 (Accession numbers: SRR25915915 to SRR25915950 for feed samples, SRR25915730 to SRR25915762 for water samples, and SRR25898824 to SRR25898859 for fish samples). Taxonomic assignment yielded 27 phyla, 52 classes, 106 orders, 145 families, and 191 genera across the whole microbiota profiles (Figure 1; Supplementary Figure 1). *Proteobacteria* (20%) was the most dominant phylum, followed by *Actinobacteria* (7%) > *Firmicutes* (6%) *> Chloroflexi* (4%) *> Bacteroidetes* (3%; Figure 1A). The γ-*Proteobacteria* (12%), followed by α-*Proteobacteria* (6%), *Bacilli* (5%), *Bacteroidia* (4%) and *Actinobacteria* (4%) occupied the five most dominant positions at the class level. The changes in the relative abundance% of the core microbes along with the growth of silver pompano are shown in Supplementary File 1. The results showed a greater abundance of core microbes in larval stages of >10 DPH than ≤10 DPH. However, the lowest abundance was noted on 10 DPH larvae. Considering all the stages (L1 to L25), the core whole microbiota occupied 58% of relative abundance and 7% of diversity at the genus level. Of these core microbes, only six ASVs (*Janthinobacterium, Pseudomonas, Solibacillus, Lysinibacillus, Peptostreptococcus,* and *Psuedoxanthomonas mexicana*) could be identified at the genus level (Supplementary File 1). The PICRUSt2 analysis showed the presence of 6,122 KEGG genes, 385 KEGG pathways, and 1,900 KEGG enzymes across the whole microbiota profiles of different ontogenetic stages of *T. blochii* (Supplementary File 2).

### Diversity measures in whole microbiota profiles across different ontogenetic stages

**Figure 2:**
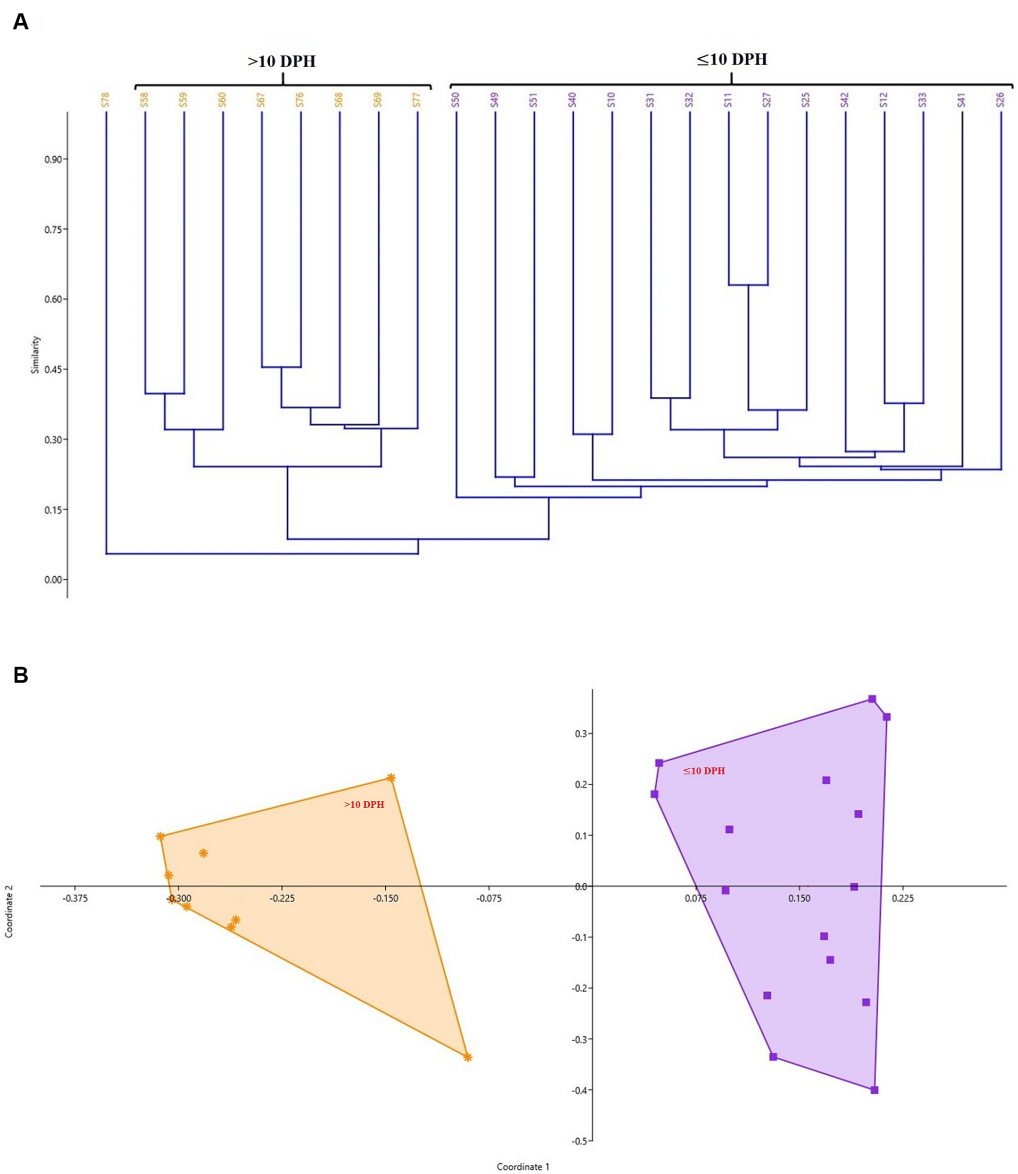
Cladogram and principal coordinate analysis based on Bray-Curtis distance metrics. **(A)** Cladogram; **(B)** Principal Coordinate Analysis (PCoA) [Cladogram and PCoA showing two well-differentiated clusters based on whole microbiota profiles (Sample ID: S78 belonging to 25 DPH remained as an outlier)]. DPH, Days post hatching; S10, S11, and S12 represent 1 DPH; S25, S26, and S27 represent 3 DPH; S31, S32, and S33 represent 5 DPH; S40, S41, and S42 represent 7 DPH; S49, S50, S51 represent 10 DPH; S58, S59, and S60 represent 12 DPH; S67, S68, and S69 represent 20 DPH, S76, S77, and S78 represent 25 DPH fish samples.

**Figure 3:**
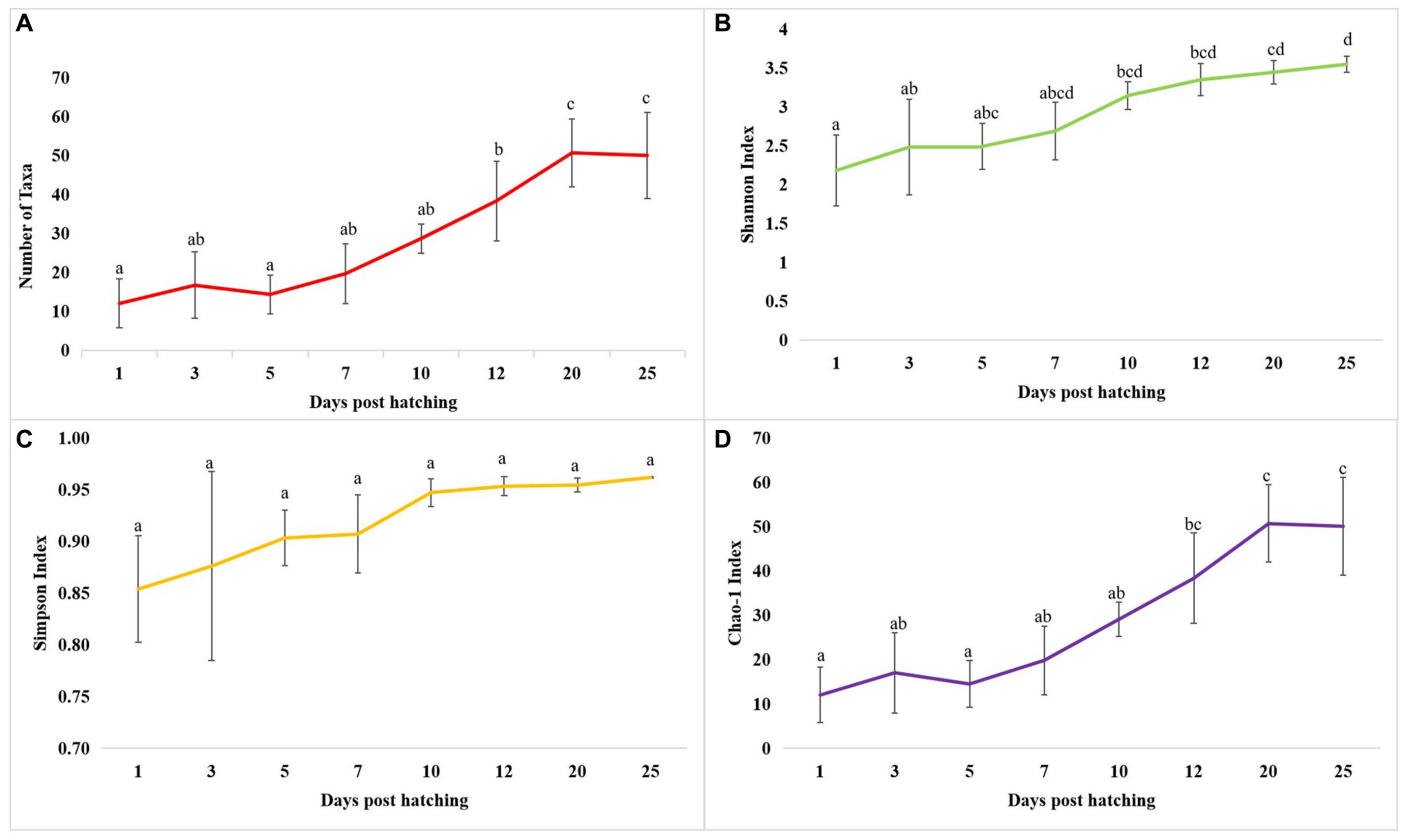
Dynamics of microbial diversity measures across *T. blochii* ontogeny. **(A)** Dynamics of number of microbial taxa; **(B)** Dynamics of number of Shannon index; **(C)** Dynamics of Simpson index; **(D)** Dynamics of Chao-1 index In all figures, different letters above the error bars indicate they are significantly different at *p* < 0.05 level.

The cladogram (Figure 2A) and PCoA (Figure 2B) analysis showed two well-differentiated clusters based on whole microbiota profiles (Supplementary Figure 2). All the samples from the larvae belonging to >10 DPH formed overlapping clusters. The larvae belonging to ≤10 DPH formed another distant cluster. The PERMANOVA test confirmed the significant difference (*p* = 0.001; *F* value: 5.89) of the clustering pattern. The α-diversity measures of ASVs also showed significant differences across the studied groups (Figure 3). Generally, larvae with >10 DPH (12, 20, 25 DPH) had higher diversity (*p* < 0.05) measures than the larvae with ≤10 DPH (1, 3, 5, 7, and 10 DPH). Further, there was a gradual increase in the diversity measures across ontogeny, which became stable from 20 DPH. More importantly, the core microbes of the whole microbiota occupied 56 and 75% of relative abundance in ≤10 DPH and > 10 DPH stages, respectively (Supplementary File 1).

### Comparison of taxonomic profiles of the whole microbiota between >10 DPH and ≤10 DPH larval stages

**Figure 4:**
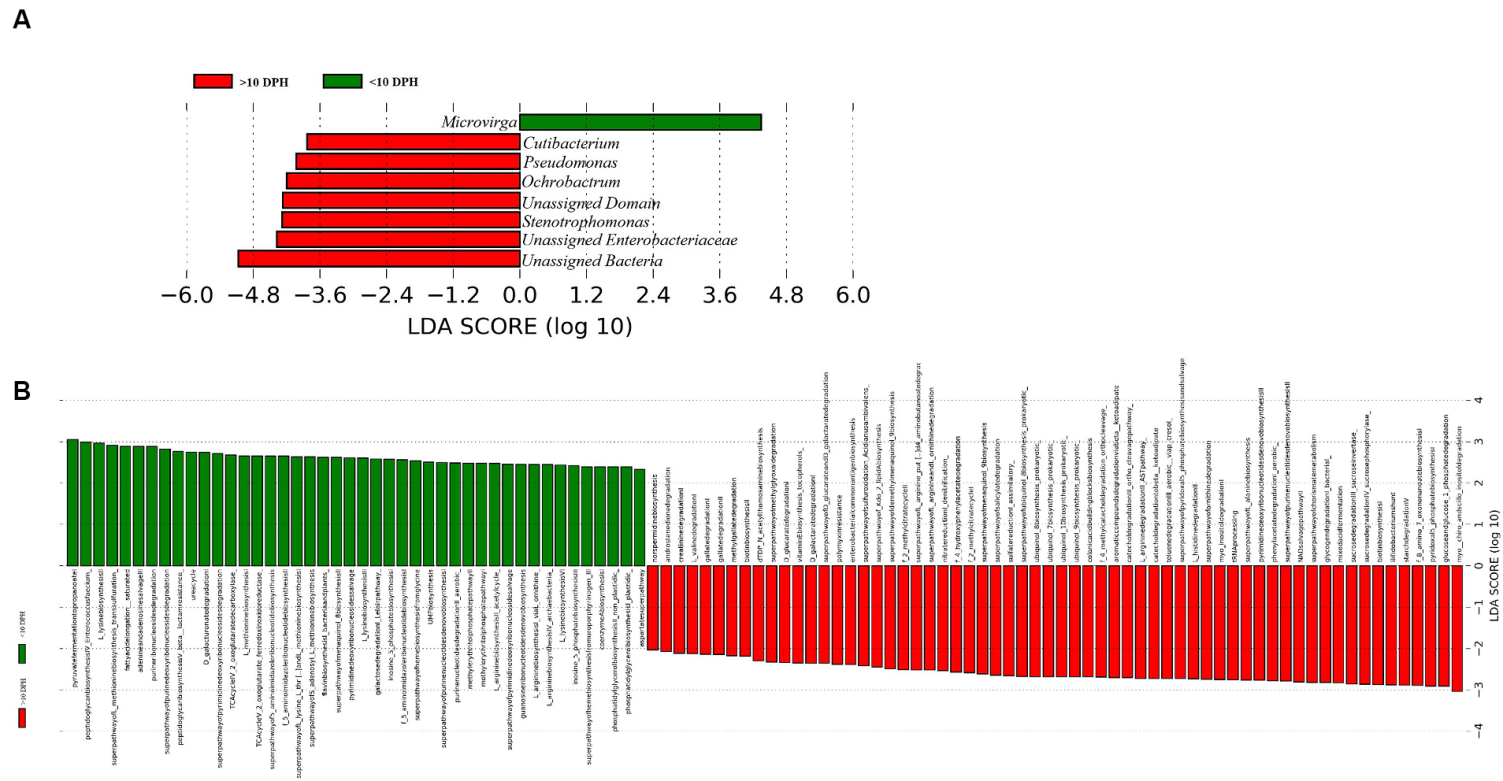
Differentially abundant microbial taxa between different ontogenetic stages of *Trachinotus blochii* larvae. **(A)** Microbial biomarkers discriminating between the ≤10 and >10 DPH whole microbiota profiles; **(B)** Discriminative KEGG pathways between the ≤10 and >10. DPH whole microbiota profiles.

*Proteobacteria* and γ*-Proteobacteria* were the most dominant phylum and class in both clusters of whole microbiota (Supplementary File 3). However, the ratio between the abundance of *Firmicutes* to *Bacteroidetes* (F/B) was significantly (*p* = 0.02) less at >10 DPH compared to ≤10 DPH (Table 1). Further, the ratio between the abundance of F/B was significantly (*p* = 0.04) decreased in larvae fed on the formulated diet (20 and 25 DPH) compared to those fed live feed (1, 3, 5, 7, 10, and 12 DPH). Further, the relative abundance of *Proteobacteria* was significantly (*p* = 0.03) less in >10 DPH than ≤10 DPH cluster (Table 1). The shared genera of both clusters occupied 50 and 75% of the relative abundance in ≤10 DPH and >10 DPH stages, respectively (Supplementary File 4). The core microbes in ≤10 DPH and >10 DPH stages are shown in Supplementary File 2. LEfSe analysis revealed eight high-dimensional differentiating microbial markers between ≤10 DPH and >10 DPH, of which the abundance of only one (*Microvirga* sp.) was significantly higher in ≤10 DPH (Figure 4A). Among the eight microbial markers, only one (*Microvirga* sp.) and three (*Pseudomonas, Unidentified Enterobacteriaceae,* and *Stenotropomonas*) were part of the core microbiota in the ≤10 DPH and >10 DPH stage, respectively. When the functional metagenomics data in the two clusters (>10 DPH and ≤10 DPH) were analyzed individually, the α-diversity measures of KEGG genes, enzymes, and pathways, except Simpson index of KEGG enzymes and Simpson index and Shannon index of KEGG pathways, were significantly higher in larvae with >10 DPH microbiota profiles than that of the larvae with ≤10 DPH (Table 1). The LEfSe analysis between the clusters showed that 105 pathways’ relative abundance significantly differed between ≤10 and >10 DPH microbiota profiles (Figure 4B). Remarkably, the relative abundances of ubiquinol biosynthetic KEGG pathways were significantly higher in >10 DPH than in ≤10 DPH.

**Table 1:** Discriminating microbial biomarkers between >10 DPH and ≤10 DPH larval stages of *Trachinotus blochii.*

Sl. no.	Criteria	≤10 DPH	>10 DPH	*p-*value
1	Relative abundance of *Proteobacteria*	22%	15%	0.038
2	F/B	1.3 ± 1.9	0.08 ± 0.257	0.026
3	α-diversity measures of KEGG genes			
	Taxa	3504.2 ± 769.83	4091.89 ± 256.02	0.014
	Simpson	1.0 ± 0.00	1.0 ± 0.00	0.001
	Shannon	7.70 ± 0.14	7.83 ± 0.06	0.004
	Chao-1	3601.13 ± 655.15	4254.44 ± 573.89	0.019
4	α-diversity measures of KEGG enzymes			
	Taxa	1239.53 ± 190.68	1393.56 ± 80.11	0.012
	Simpson	1.0 ± 0.00	1.0 ± 0.00	0.028
	Shannon	6.67 ± 0.09	6.76 ± 0.05	0.006
	Chao-1	1274.40 ± 152.75	1415.44 ± 100.49	0.012
5	α-diversity measures of KEGG pathways			
	Taxa	251 ± 40.23	286.44 ± 16.67	0.007
	Simpson	0.99 ± 0.00	0.99 ± 0.00	0.018
	Shannon	5.33 ± 0.11	5.42 ± 0.04	0.012
	Chao-1	251.07 ± 40.08	286.67 ± 16.70	0.007

F/B, Ratio between the abundance of *Firmicutes* to *Bacteroidetes*; DPH, Days post hatching; KEGG, Kyoto Encyclopedia of Genes and Genomes.

### Gut microbiota profiles of *Trachinotus blochii* fingerlings

**Figure 5:**
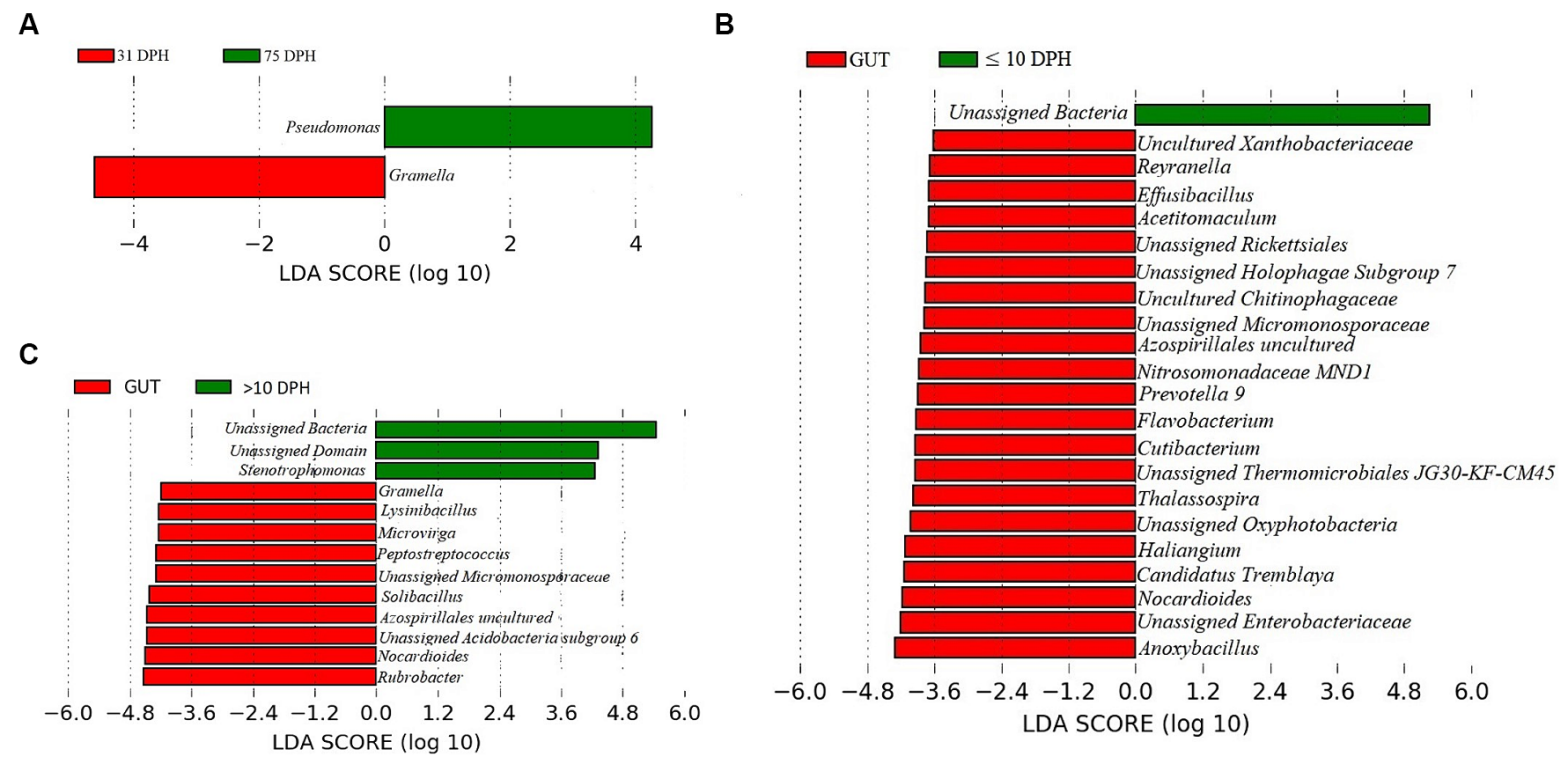
Differentially abundant taxa in the gut microbiota between different ontogenetic stages of *Trachinotus blochii* and differentially abundant taxa between gut microbiota and whole larval microbiota. **(A)** Microbial biomarkers discriminating between the 31 and 75 DPH gut microbiota profiles; **(B)** Microbial biomarkers discriminating between the gut microbiota (combined data of 31 and 75 DPH gut microbiota) and ≤10 DPH whole microbiota profiles; **(C)** Microbial biomarkers discriminating between the gut microbiota (combined data of 31 and 75 DPH gut microbiota) and >10 DPH whole microbiota profiles.

Analysis of α-diversity measures of ASVs in the gut showed that the value of Shannon’s index varied from 3.08 to 3.9, indicating high diversity. Simpson richness ranged from 0.95 to 0.97, and evenness varied from 0.74 to 0.85, signifying high richness and high evenness, respectively. There was no statistically significant difference in the α-biodiversity indices between 31 and 75 DPH gut (*p* > 0.05). PERMANOVA analysis showed no significant difference between 31 and 75 DPH (*p* = 0.1). There were 18 phyla, 45 classes, 77 orders, 100 families, and 121 genera. The most dominant phylum was *Proteobacteria* (33%), followed by *Actinobacteria* (17%) and *Firmicutes* (12%; Figure 1A; Supplementary File 3). The four most abundant genera in the overall gut microbiota were *Janthinobacterium*, *Nocardioides, Solibacillus*, and *Rubrobacter* (Figure 1B). The core gut microbiota occupied 54% of relative abundance (Supplementary File 2). Of the core microbes, only seven ASVs (*Janthinobacterium, Pseudomonas, Solibacillus, Cutibacterium, Peptostreptococcus, Candidatus Tremblaya,* and *Lysinibacillus*) could be identified up to the genus level. The comparison of gut microbiota profiles between 31 and 75 DPH revealed that the relative abundance of none of the phyla was significantly altered between each other. Nevertheless, at the class level, a significant decrease (~10 times) in *Clostridia* was observed in 75 DPH gut. LEfSe showed only two discriminating genera between these two ontogenies (Figure 5A). The relative abundance of *Pseudomonas* spp. was significantly increased, and *Gramella* was significantly decreased in 75 DPH than 31 DPH. When the core microbiota and LEfSe results were compared, both differentiating markers were part of core gut microbial biomarkers. Analysis of shared features between the gut and whole microbiota showed that the shared genera occupied 47% of relative abundance in the gut (Supplementary File 4). Regarding functional metagenomics, the gut had 5,900 KEGG genes, 379 KEGG pathways, and 1,881 KEGG enzymes (Supplementary File 2). Further, Shannon’s diversity index of KEGG enzymes was found to be significantly increased at 75 DPH compared to 31 DPH gut (*p* < 0.05). The LEfSe showed 22 and 13 discriminating genera between the gut with ≤10 DPH (Figure 5B) and >10 DPH whole larval microbiota, respectively (Figure 5C).

### Egg microbiota composition in comparison to the whole larval and gut microbiota

**Figure 6:**
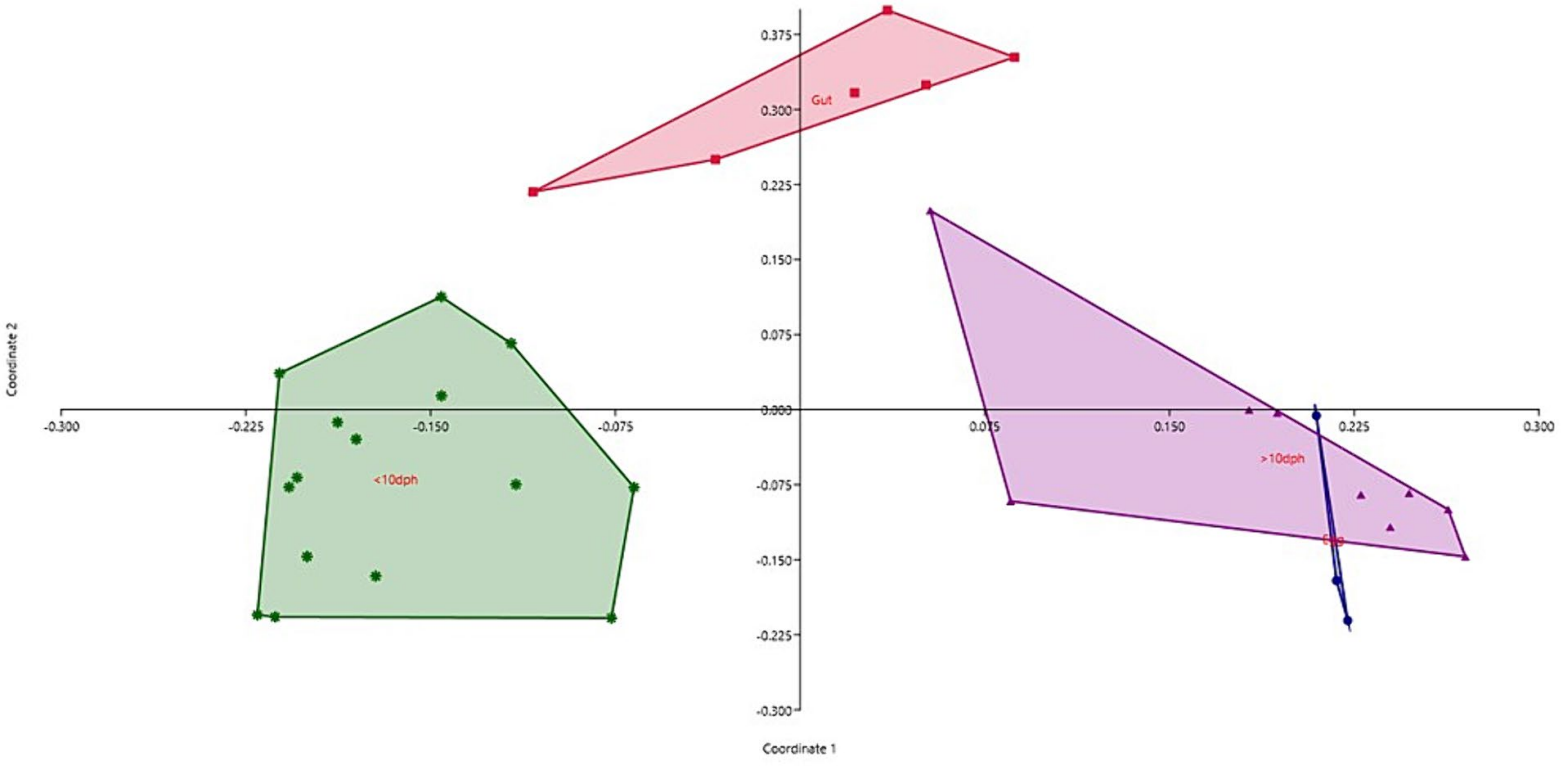
Principal coordinate analysis plot between different ontogenetic stages of *Trachinotus blochii* based on Bray-Curtis distance metrics. The analysis shows the distinct bacterial communities between different ontogeny. DPH, Days post hatching.

**Figure 7:**
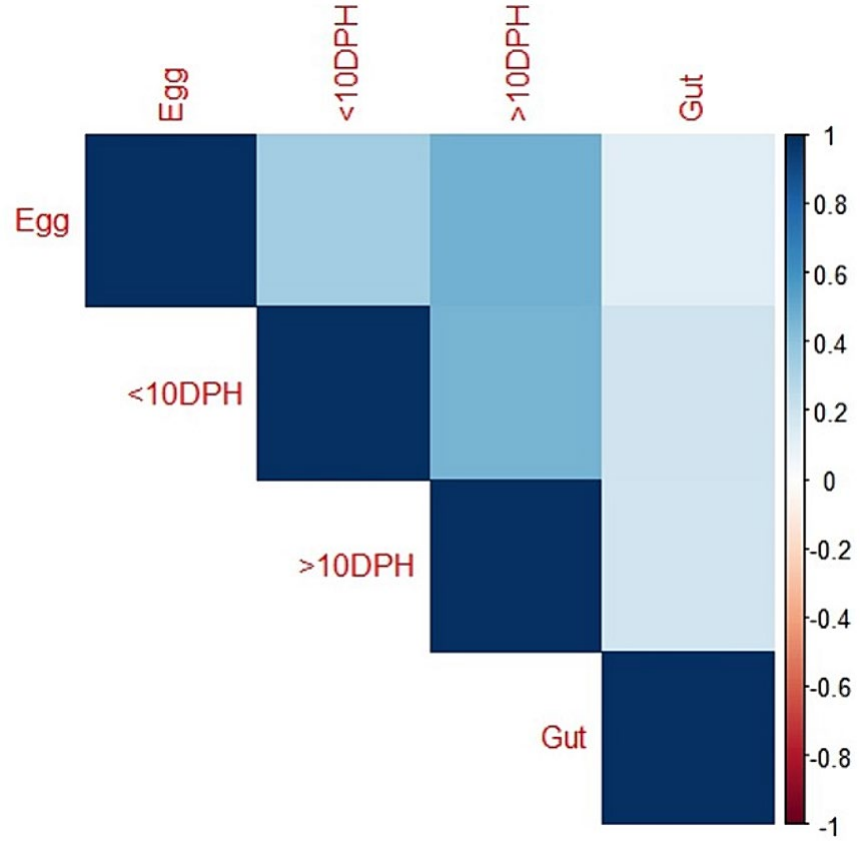
Correlation analysis of the egg microbiota with different ontogenetic stages of *Trachinotus blochii.*

Taxonomic assignment yielded 16 phyla, 33 classes, 59 orders, 74 families, and 84 genera in the egg microbiota profiles of *T. blochii* (Figure 1; Supplementary Figure 1). *Proteobacteria* (24.8%) was the most dominant phylum, followed by *Actinobacteria* (5.04%), *Cyanobacteria* (4.17%), *Chloroflexi* (3.76%), *Bacteroidetes* (3.05%), and *Firmicutes* (2.03%). The γ-*Proteobacteria*, followed by α-*Proteobacteria*, *Actinobacteria,* and *Bacilli* occupied the four most dominant positions at the class level. The core microbiota in the egg contained 12 genera occupying ~76% relative abundance (Supplementary File 1). The PCoA followed by PERMANOVA identified significant (*p* = 0.001) four clusters based on microbiota (Figure 6). The egg microbiota communities formed an overlapping clustering pattern with >10 DPH microbiota. The *F*-values revealed that egg microbiota had more similarity to >10 DPH (*F* = 1.48; *p* = 0.03) followed by gut (*F* = 2.51; *p* = 0.01) then ≤10 DPH (*F* = 3.63; *p* = 0.002) microbiotas, which were confirmed through correlation analysis between the ASV features of these three groups (Figure 7). Comparison of egg microbiota to whole larval (≤ 10 DPH and >10 DPH) and gut microbiota indicated no significant difference in the ratio between the abundance of *Firmicutes* to *Bacteroidetes* among different groups. The ratio between *Proteobacteria* to *Bacteroidetes* was significantly higher in the egg microbiota. At the phyla level, the abundances of *Epsilonbacteraeota and Tenericutes* were significantly higher in the egg microbiota than in the other three microbiota profiles. The PERMANOVA analyses demonstrated significant differences between groups (Supplementary Table 2). Further, the results signified the higher similarity of the egg microbiota to >10 DPH whole microbiota (*F* = 1.47; *p =* 0.03) followed by the gut microbiota (*F* = 2.5; *p* = 0.013). The shared genera from the egg microbiota occupied 58, 81, and 36% relative abundance in ≤10 DPH, >10 DPH, and gut microbiota, respectively (Supplementary File 4).

The LEfSe analysis of different clusters indicated significantly higher abundance of 54 genus-level taxa among the egg microbiota than ≤10 DPH stages, with the abundances of *Marinobacter* and *Stenotrophomonas* being the most differentiating microbial biomarkers (Supplementary Figure 3). Among the 54 microbial markers, five (*Stenotrophomonas, Marinobacter, Ochrobactrum,* Uncultured *Anaerolineaceae,* and Unidentified *Chloroflexi*) were part of the core microbiota in the egg. The LEfSe analysis between the egg microbiota and >10 DPH larval microbiota showed six genus-level taxa as high dimensional differentiating microbial markers, of which abundances of five and one were significantly increased and decreased, respectively, in the egg microbiota. Among the six microbial markers, five (*Stenotrophomonas, Rubrobacter, Marinobacter, Burkholderia-Caballeronia-Paraburkholderia,* and Unidentified *Chloroflexi*) were part of the core egg microbiota. The LEfSe showed five discriminating genera between the egg and gut microbiota, with a higher abundance of *Marinobacter, Stenotrophomonas,* and *Paraburkholderia* in the egg (Supplementary Figure 3).

### Microbiota in microalgae, rotifer, artemia, formulated feed, and rearing water

**Figure 8:**
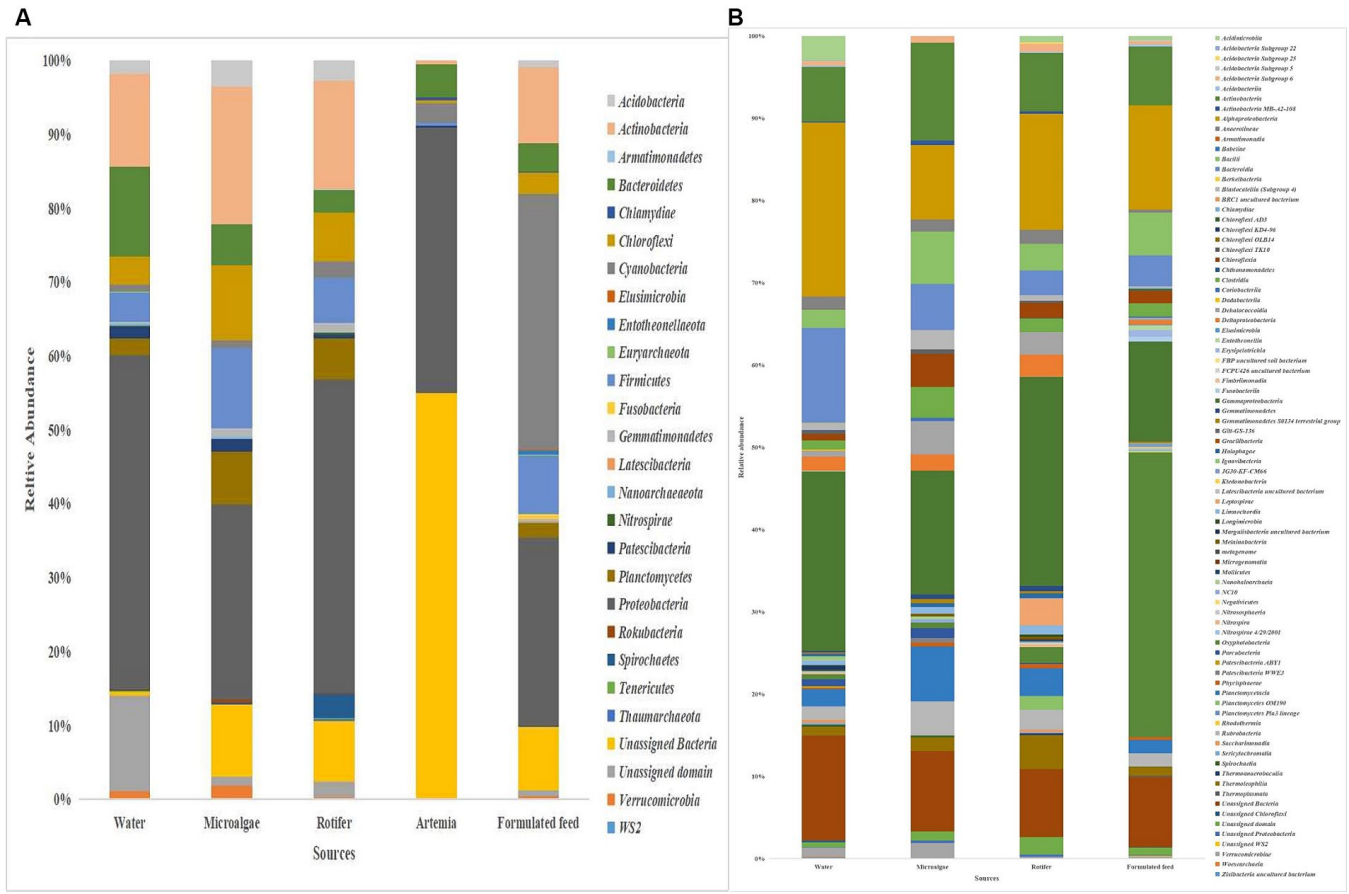
Taxonomic landscape in the microbiota profiles of the microalgae, rotifer, artemia, formulated feed, and rearing water. Taxa representing >0.1% of total abundance were only considered for making plots. For genus-level data, only the top 50 genera were used for plotting. **(A)** At phylum level; **(B)** At genus level. DPH, Days post-hatching.

*Proteobacteria* was the most dominant phylum in microalgae (26%), rotifer (42%), artemia (36%), and rearing water (45%), followed by *Actinobacteria* in microalgae (19%), rotifer (15%), and water (12%; Figure 8). *Bacteroidetes* formed the second position in artemia (5%). In formulated feed, *Cyanobacteria* (35%), followed by *Proteobacteria* (26%), and *Actinobacteria* (10%) occupied the three most dominant positions. Of the 106 genera in microalgal microbiota (Supplementary Figure 4), 23 were shared with rotifer, representing 56% of total abundance in the rotifer (Supplementary File 5). Analysis of sharing genera showed that the microalgal microbiota occupied 75, 81, and 53% of relative abundance in ≤10 DPH, >10 DPH and gut, respectively. In comparison, the rotifer microbiota occupied 74%, 81%, and 52% abundance in ≤10 DPH, >10 DPH, and gut. The microbiota of artemia occupied 45%, 64%, and 11% abundance in ≤10 DPH, >10 DPH, and gut. The water microbiota occupied 58%, 66%, 83%, and 30% abundance in egg, ≤10 DPH, >10 DPH, and gut, respectively (Supplementary File 5).

### Comparison of environmental microbiota with whole microbiota profiles of *Trachinotus blochii* larvae

**Figure 9:**
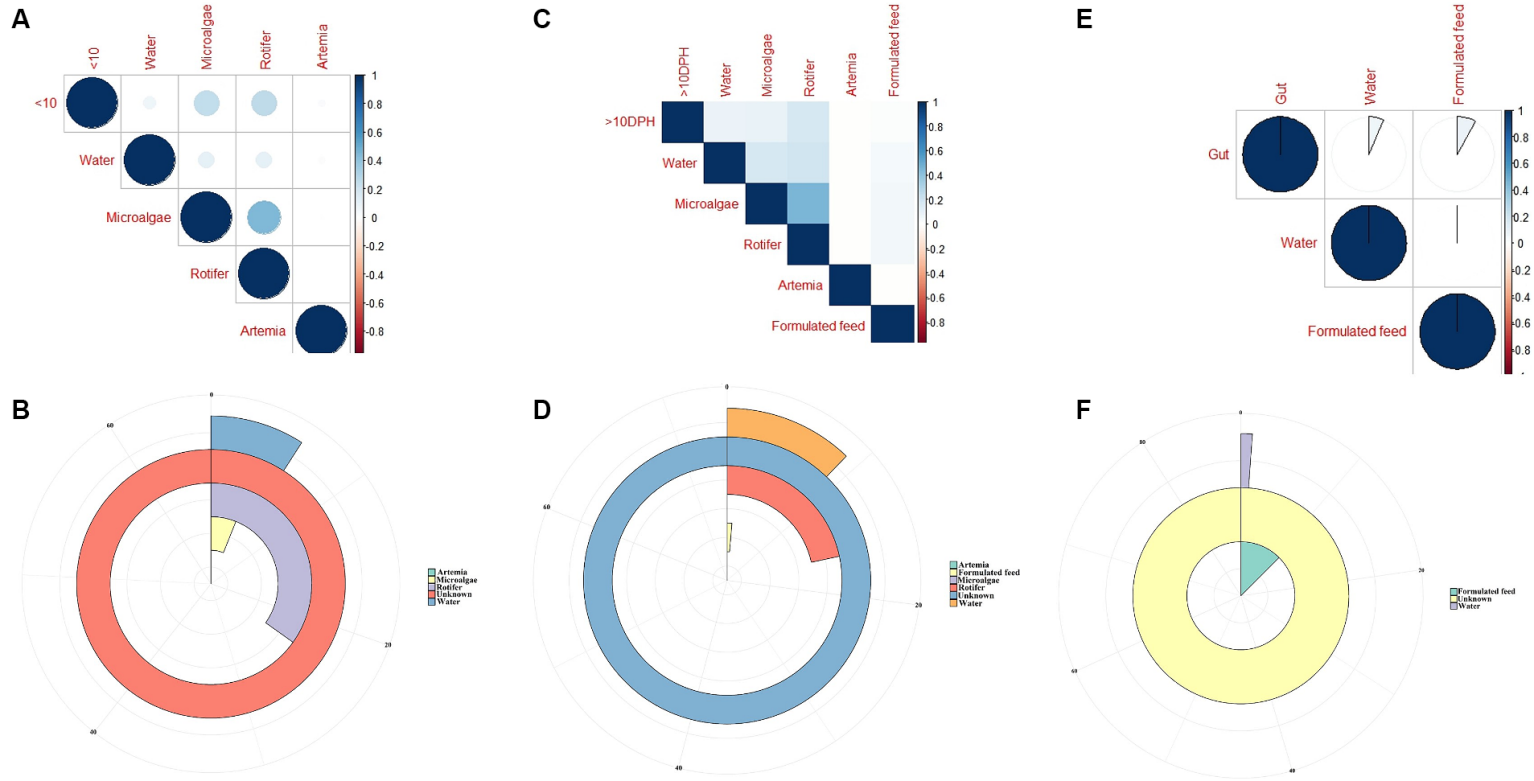
Results of correlation analysis and “Feast program”-based prediction between environmental microbiota and the microbiota different ontogenetic stages of *Trachinotus blochii*. **(A)** Correlation analysis between the ASV features of ≤10 DPH whole microbiota and microbiota of ecological factors; **(B)** Predictions using the “Feast program” on ≤10 DPH whole microbiota; **(C)** Correlation analysis between the ASV features of >10 DPH whole microbiota and microbiota of ecological factors; **(D)** Predictions using the “Feast program” on >10 DPH whole microbiota; **(E)** Correlation analysis between the ASV features of gut microbiota and microbiota of ecological factors; **(F)** Predictions using the “Feast program” on gut microbiota.

The *F*-values in the PERMANOVA revealed that whole larval microbiota had more similarity to microalgal microbiota (*F* = 3.53; *p* = 0.002) followed by rotifer (*F* = 5.7; *p* = 0.0001), artemia (*F* = 5.88; *p* = 0.0006) and water microbiota (*F* = 10.75; *p* = 0.0001). The correlation analysis showed that ≤10 DPH microbiota had significant positive correlations with microalgal and rotifer microbiota (r = 0.2; *p < 0.001*) followed by water and then artemia (Figure 9A). The “Feast” predictions determined that rotifer, followed by microalgae, then water, and artemia contributed toward the whole microbiota profiles of ≤10 DPH (Figure 9B).

The PERMANOVA showed the maximum similarity of >10 DPH microbiota to the microalgae (*F* = 3.5; *p* = 0.0001) followed by rotifer (*F* = 4.2; *p* = 0.0001), water (*F* = 5.88; *p* = 0.0003) and artemia microbiota (*F* = 11.4; *p* = 0.003). The maximum correlation was observed with rotifer (Figure 9C). The “Feast predictions” determined rotifer contributed maximum in >10 DPH (Figure 9D), even though contributions from rotifer and microalgae were less than ≤10 DPH (Figure 9B). Further, the combined effect of different horizontal routes was more in ≤10 DPH (~27%) compared to <10 DPH (~17%). However, it is essential to note that >50% of microbes in ≤10 DPH and >10 DPH were from unknown or unsampled sources (Figure 9D).

### Comparison of environmental microbiota with the gut microbiota profiles

The PERMANOVA results revealed that the gut microbiota had more similarity to the water microbiota (*F* = 1.95; *p* = 0.009) followed by the formulated feed (*F* = 3.15; *p* = 0.001). However, the correlations were almost similar with water and feed microbiota (r = 0.08 and 0.06; *p < 0.001*; Figure 9E). The “Feast predictions” determined that the feed contributed maximum toward the gut microbiota followed by water (Figure 9F).

**Figure 10:**
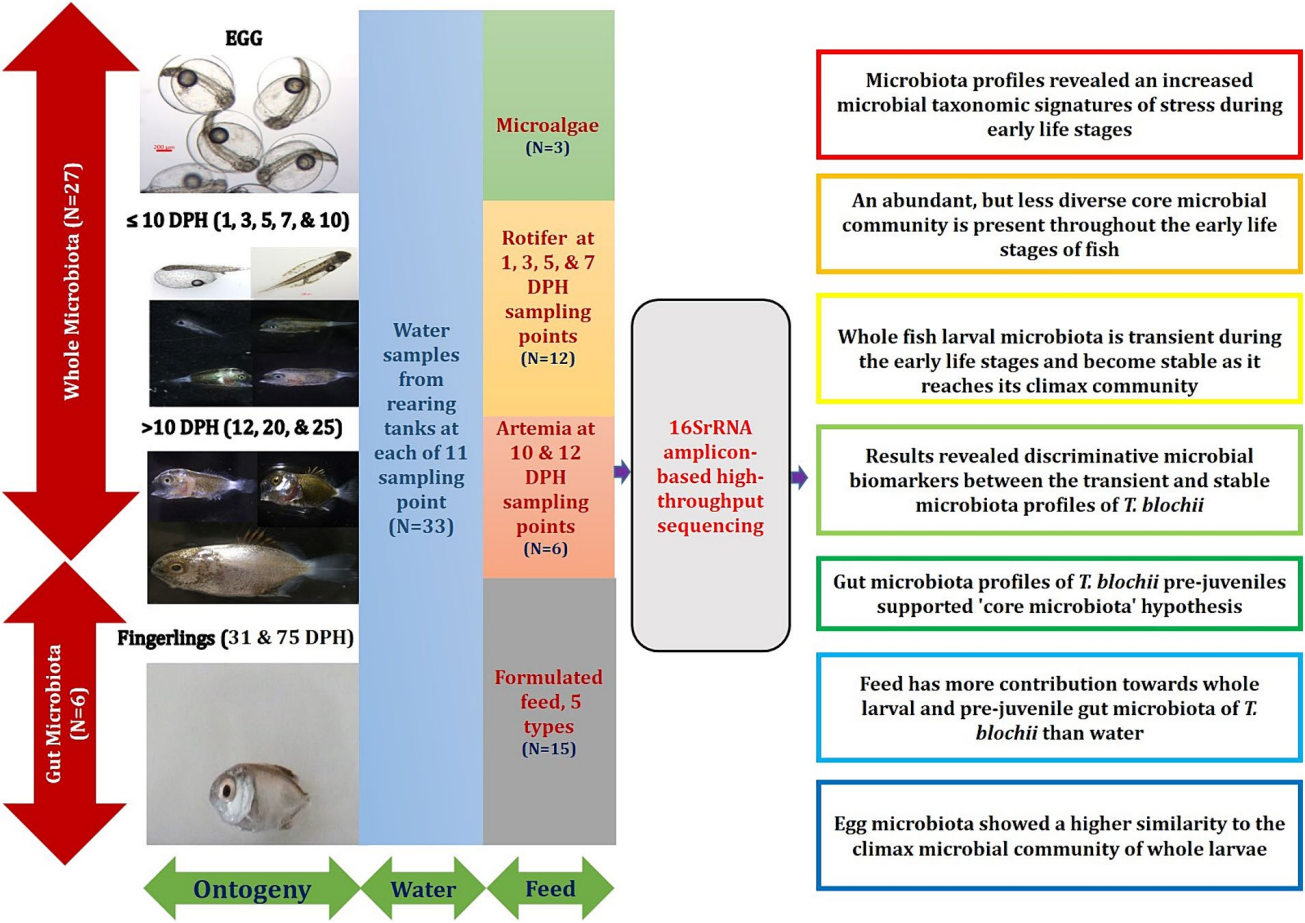
Overall experimental design and the major findings of the present study.

The overall experimental design and the major findings of the present study is depicted in Figure 10.

## Discussion

### An abundant but less diverse core microbial community is present throughout the early life stages of fish

The core microbes occupied only ~7% of the diversity at genus level, but represented ~59% of ASV abundance, reinforcing the previous hypothesis that a small but abundant core microbiota community is present in the early life stages of fish (Borges et al., 2021). Existence of a small core set of microbial taxa that persists across the whole life of a parasite is reported in a helminth parasitic trematode, *Coitocaecum parvum* (Jorge et al., 2020). Studies identifying the core microbiota of fish larvae are very scarce, except one study, which revealed that the *S. lalandi* core microbiota comprised ~56% of the total relative read abundance (Walburn et al., 2019), similar to our observation. However, the core microbes from *T. blochii* differed from those reported for *S. lalandi*. Of the core microbes of *T. blochii*, six were identified as *Janthinobacterium, Pseudomonas, Solibacillus, Lysinibacillus, Peptostreptococcus,* and *Psuedoxanthomonas mexicana*. Of these, *Janthinobacterium, Pseudomonas, and Lysinibacillus,* were reported to contain probiotics that can prevent fish diseases (Hornung et al., 2013; Asencio et al., 2014; Eissa et al., 2014; Liu et al., 2015; Wang et al., 2018). Interestingly, Liu et al. (2015) demonstrated that the live inoculum of *Pseudomonas* sp. could provide new biological and sustainable means to mitigate many fish bacterial diseases. Further, transportation stress significantly decreased the abundance of *Pseudomonas* and *Lysinibacillus* in cobia larvae, indicating a possible beneficial role of these genera in fish larvae (Sumithra et al., 2022a). In short, even though the functional role of the core microbes of *T. blochii* remains unclear, their conspicuous presence throughout different ontogenetic stages warrants future investigation into their role in larval health.

### Microbiota profiles revealed increased levels of microbial taxonomic signatures of stress during early life stages

Studies on the larval microbiota of *Gadus morhua* and *Kryptolebias marmoratus* (Bakke et al., 2015; Forberg et al., 2016) showed changes in the microbial community along with the ontogenetic progression and shed light on the importance of early life communities of the fish. Analysis of the β-diversity measures from 1 to 25 DPH whole microbiota showed two well-differentiated clusters, with one cluster comprising ≤10 DPH larvae and another cluster formed by >10 DPH larvae. Further, the samples belonging to ≤10 DPH had significantly lower α-diversity measures than larvae with >10 DPH. Additionally, within the ≤10 DPH cluster, larvae from 1 and 3 DPH had significantly lower (*p* < 0.05) α-diversity measures than all other groups. In other words, the bacterial richness and diversity measures significantly increased across the ontogenetic progression till 12 DPH and then remained stable from 12 to 25 DPH, with no significant difference between 12 and 25 DPH groups. The pattern of α-diversity measures, evidenced by increasing up to a particular stage, and thereafter remaining stable, was reported in the early-stage microbiota of Yellowtail Kingfish (*S. lalandi*; Walburn et al., 2019). Egerton et al. (2018) also reported that the richness and diversity of bacteria increase as the fish grows. Similar to taxonomic metagenomic measures, the diversity measures of functional metagenomics were significantly higher in larvae with >10 DPH than that of the larvae with ≤10 DPH. Conversely, this trend was not observed in freshwater channel catfish, where no differences between fish age groups were found owing to high interindividual variation (Bledsoe et al., 2016). The mammalian microbiota studies revealed that a greater microbial diversity conferred a beneficial effect on the host, whereas the lowered microbial diversity has been associated with several stressors (Rodríguez et al., 2015). Accordingly, significantly lower bacterial diversity measures in the earlier life stages of the fish may reflect increased stress during the initial days. Another proposed biomarker for microbial dysbiosis and stress in avian and mammalian microbiota are the increased ratio of the relative abundance between the *Firmicutes* to *Bacteroidetes* (F/B; Liu et al., 2020). A significant increase in F/B was reported in *Colossoma macropomum* (Sylvain et al., 2016) and cobia larvae (Sumithra et al., 2022a) following acid stress and transportation, respectively. Strikingly, the F/B ratio was significantly higher in ≤10 DPH larvae. Further, the ratio was significantly less in the larvae fed with formulated feed. Even though further studies are needed to confirm the observed trends across different ontogenetic stages of *T. blochii,* the two proposed microbial taxonomic signatures of stress explained above indicated increased stress during early life stages, which can serve as a novel explanation for increased mortality rates reported in ≤10 DPH larvae of *T. blochii* (Abdul Nazar et al., 2012).

### The whole larval microbiota is transient during the early life stages and becomes stable as it reaches its climax community

As discussed earlier, α-diversity measures of early microbiota of *T. blochii* significantly increased across the ontogenetic progression, thereafter remained stable from 20 DPH. Further, the core microbiota occupied ~50% and ~80% ASV abundance in ≤10 DPH and >10 DPH. The core microbes were reported as stable microbiota members and increased while reaching the climax community (Berg et al., 2020). Therefore, the observations of the higher abundance of transient microbes during ≤10 DPH suggested that the whole microbiota of *T. blochii* becomes comparatively stable in later life stages. Similar to our observations, achieving a relatively stable gut microbiota after the initial days of life has been reported in certain fish species (McIntosh et al., 2008; Larsen et al., 2014; Grande Burgos et al., 2018; Rudi et al., 2018; Zhang et al., 2018), even though the corresponding data on the whole microbiota is completely lacking. However, it is hypothesized that the fish larval microbiota changes faster during early life stages until metamorphosis due to the changes in the host-microbe and microbe-microbe interactions, and our results supported this hypothesis (Vadstein et al., 2018). Briefly, the higher abundance of core microbes in >10 DPH and results of α -diversity measures of taxonomic and functional metagenomics propel us to make a new hypothesis that the whole fish larval microbiota is highly transient during the early life stages and becomes stable as it reaches its climax community. Further, based on the results, we hypothesize that the best time to influence the larval microbiota in marine teleost is ≤10 DPH.

### Discriminative microbial biomarkers between the transient and stable microbiota profiles of *Trachinotus blochii*

The linear discriminant analysis effect size (LEfSe) revealed eight high-dimensional differentiating markers between the two identified ontogenetic clusters. *Microvirga* spp. was increased and consistently present in all ≤10 DPH samples. *Microvirga* species are ubiquitous in soil and water microbiota (Msaddak et al., 2019). The increased and consistent presence of *Microvirga* spp. in the early larval stages suggested a stronger influence of environment in ≤10 DPH. Further, the increased abundance of *Pseudomonas* spp. a reported fish probiotic microbe (Abd El-Rhman et al., 2009; Liu et al., 2015) in later stages might be an adaption toward establishing beneficial strains along the development.

The LEfSe analysis also demonstrated 105 differentiating KEGG pathways between ≤10 and >10 DPH. More importantly, the ubiquinol biosynthetic pathways were significantly higher in >10 DPH than in ≤10 DPH. As the pathways related to ubiquinol biosynthesis had positive associations with increased general health (Gacesa et al., 2022), the increased relative abundance in >10 DPH might indicate greater larval well-being. A noteworthy discriminating feature between the two ontogenetic clusters was the significant decrease of *Proteobacteria* in >10DPH larvae. Our observations are consistent with Sylvain and Derome (2017), who documented an increased *Proteobacteria* in the gut microbiota of early-stage zebrafish larvae than in later stages. The explanation given by them was that several opportunistic strains in *Proteobacteria* colonize the fish during early development, which become less abundant as the resistance increases during development. The assumption was further strengthened by the observed 10 times decrease in *Vibrionaceae* (which contain many marine pathogenic strains) in >10 DPH than ≤10 DPH of the present study. Altogether, the discriminative analysis of the two identified whole microbiota clusters suggested an adaptive mechanism for establishing beneficial strains along the ontogenetic progression.

### Gut microbiota profiles of *Trachinotus blochii* fingerlings supported the “core microbiota” hypothesis

The core gut microbiota occupied 54% relative abundance considering the data from 31 and 75 DPH. As per the previous observations in different fishes, the core gut microbiota makes up ~60% of the total gut ASVs (Sullam et al., 2012; Li et al., 2020), supporting our results. The results suggest that an abundant core microbiota can persist in the fish gut, supporting the “core microbiota” hypothesis (Roeselers et al., 2011). To identify the possible progressive transition of gut microbiota across ontogeny, 31 and 75 DPH were compared. However, there was no significant difference between these groups regarding both α and β diversity indices. Further, none of the phyla was significantly altered between each other, suggesting that the gut microbes are much more stable even from the first month of hatching, provided the ecotype remained same. In support of our results, microbial communities inhabiting the intestines of catfish became relatively stabilized from 3 months of age (Bledsoe et al., 2016). Xie et al. (2021) reported that the α-diversity indices of intestinal microbiota were decreased along with the growth of *Procambarus clarkia* with maximum diversity in the larval stages and lowest in the adult stages. However, they have not examined the difference within adult or juvenile stages of fish, so that direct comparison with the present study was not possible. The present study indicated a significant decrease (~10 times) in *Clostridia* at the class level in 75 DPH gut compared to 31 DPH. *Clostridia* is reported as the gut microbial flora in different marine herbivorous fish species (Clements et al., 2007). The reduction in their relative abundance toward ontogenic progression may be an adaptation toward carnivorous feeding behavior that demands future research. LEfSe analysis showed two discriminating genera between 31 and 75 DPH gut microbiota, as the relative abundance of *Pseudomonas* spp. was significantly increased, and *Gramella* was significantly decreased in 75 DPH than the 31 DPH. When the core microbiota and LEfSe results were compared, both differentiating markers were part of core gut microbial biomarkers. The presence of *Gramella* in the fish gut was not reported earlier. *Pseudomonas* is a frequently reported gut microbiota member in carnivorous fish (Egerton et al., 2018) and contains many promising probiotic strains. The increase in their abundance toward ontogenic progression may be an adaptation toward establishing beneficial strains. The hypothesis was further strengthened by the results on higher diversity measures of KEGG enzymes in 75 DPH than in 32 DPH, as greater microbial diversity benefits the host (Rodríguez et al., 2015).

### The egg microbiota showed a higher similarity to the climax microbial community of whole larvae

The egg microbiota showed a higher similarity to >10 DPH microbiota and the gut microbiota compared to ≤10 DPH microbiota. Additionally, the shared genera from the egg microbiota occupied 58, 81, and 36% of relative abundance in ≤10 DPH, >10 DPH, and the gut microbiota, respectively. The lesser shared features with the gut microbiota were because the gut represents only a portion of the total microbiota (Legrand et al., 2020). The correlation analysis verified these results, as the highest correlation was recorded between egg microbiota and >10 DPH larval microbiota. The fertilized egg microbiota was reported to colonize in the growing embryo and, subsequently, the fish larvae (Ganal et al., 2011), which might be responsible for the observed similarity of egg microbiota on subsequent ontogenetic stages. However, the more significant correlation with the climax community of whole larvae was surprising and warrants future investigation. Noteworthily there was a significant positive correlation between the gut microbiota and egg microbiota, which supports the earlier hypothesis that the egg microbiota can crucially contribute to the gut microbial community structure in fishes, along with the rearing water and live feed microbiota (Sullam et al., 2012). The results also open up the possibilities for vertical symbiont transmission in fish to the climax community of whole larvae and fish gut, as hypothesized by Borges et al. (2021). The function of vertically transmitted microbiotas is an interesting future research topic and requires the application of meta transcriptomics. Briefly, the data suggested that microbiota community assembly in fish’s early life stages is achieved through vertical and lateral symbiont transmission.

### Feed contributes more toward the whole larval and fingerling gut microbiota of *Trachinotus blochii* than water

The current debate concerns the connectivity between fish larvae, feed, and water microbiotas in the complex fish larviculture ecosystem (Borges et al., 2021). To investigate the contribution of these factors in shaping marine fish larval and gut microbiota, a three-independent analysis, *viz.* pairwise comparison after One-way PERMANOVA analysis, correlation analysis between ASV features, and predictions using the “Feast microbial source tracking program” was performed. All three analyses showed that water and feed-associated microbiota significantly contributed to the whole larval and fingerling gut microbiota. More importantly, all the analyses unambiguously showed that the feed (including microalgae, rotifer, artemia, and formulated feed) contributed more to fish microbiota than water. Previous studies investigating the marine fish larval microbes in comparison to their rearing systems through culture-based approaches and 16S rRNA amplicon-based fingerprinting methods showed that the rearing water microbes had a critical role in the larval microbiota (Sun et al., 2013; Vadstein et al., 2018). However, the data obtained using such approaches is limited. Only a few studies used omics-based methodologies to investigate the contribution of feed and water on the teleost microbiota. Bledsoe et al. (2016) and Califano et al. (2017) demonstrated that water contributed less to the larval microbiota in parallel to our observations. In contrast, Walburn et al. (2019) showed the strong influence of the water microbiota on the larval microbiota. The analysis between the statistically differentiated clusters of whole larval microbiota in the present study showed that even though the feed microbiota contributed more to ≤10 DPH stages, the rearing water caused more contribution to >10 DPH than feed. Combined, these different observations suggest that both water and feed can contribute to early microbiota development, and the relative contribution of either factor may depend on the fish species and stages, reinforcing the importance of species-specific and stage-specific studies. Combined, these different observations suggest that both water and feed can contribute to early microbiota development, and the relative contribution of either factor may depend on the fish species, reinforcing the importance of species-specific studies. Our results also suggested that rotifer enrichment is the optimal medium for microbiota manipulation in marine fish larvae. However, the artemia microbiota had only a negligible contribution to larvae, supporting the observations made by Walburn et al. (2019). Noteworthily, >50% of whole larval and gut microbes were from unknown sources. Supporting our observation, previous research in different fish species reported that several larval microbes are dissimilar from rearing water and live-feed microbes, and are likely to be host-species specific (Bakke et al., 2015; Califano et al., 2017; Zhang et al., 2018; Borges et al., 2021). The results also warrant future research on the contribution of milt and roe microbiota on the microbiota of later ontogenetic stages of fish.

The development of sustainable microbiota manipulation applications in the fish industry depends on an integrated knowledge of the various driving forces (abiotic and biotic factors) in establishing microbial associations (Derome and Filteau, 2020). The present study showed that microbiota community assembly in the early life stages of *T. blochii* is achieved through vertical (egg microbiota) and horizontal (water and feed) transmission. However, the horizontal route contributed more to the earlier (≤10 DPH) life stages. Within the horizontal routes, we have examined the relative effect of feed and water, the two major focus points for microbiota manipulation. The results showed that the horizontal transmission of microbial symbionts mainly occurs through feed followed by water, helping to shape the taxonomic structure of the whole larval and gut microbiota. Further, results suggested that rotifer will be the optimal medium for microbiota manipulation in *T. blochii* larval microbiota manipulation.

## Conclusion

The present study generates first-hand insights and in-depth information on the composition, dynamics, and ecological contributions to the whole microbiota in the early life stages (from fertilized eggs till the completion of metamorphosis) and the gut microbiota of fingerlings in one promising tropical marine food fish species, *T. blochii*, throwing several thought-provoking insights into beneficial microbial management strategies for sustainable mariculture practices. Both whole larval and gut microbiota profiles supported the “core microbiota” hypothesis of teleost. The results suggested that the whole fish larval microbiota is transient during the early life stages (≤10 DPH) and becomes stable as it reaches its climax community, opening up better opportunities for microbiota manipulation at ≤10 DPH. Further, the results pointed out the critical discriminating metagenomic signatures between different ontogenetic life stages of snub nose pompano microbiota. Several factors, including feed, rearing water, egg, and fish species have significantly affected microbial community composition, with feed contributing more than water. The results also suggested that feed especially rotifers will be the optimal media for a microbe-based management strategy. Briefly, the data generated from this research can be used to complement the teleost microbiota research, particularly for applying suitable microbiota intervention strategies during marine larviculture practices, where the maximum mortality rates are encountered.

## Data availability statement

The datasets presented in this study can be found in online repositories. The names of the repository/repositories and accession number(s) can be found in the article/Supplementary material.

## Ethics statement

The animal study was approved by ICAR-CMFRI, Kochi, India (BT/AAQ/3/SP28267/2018). The study was conducted in accordance with the local legislation and institutional requirements.

## Author contributions

TS: Conceptualization, Formal analysis, Investigation, Methodology, Supervision, Visualization, Writing – original draft. SSh: Conceptualization, Funding acquisition, Project administration, Supervision, Visualization, Writing – review & editing. GS: Data curation, Formal analysis, Methodology, Software, Writing – original draft. APG: Methodology, Resources, Supervision, Writing – review & editing. SSu: Methodology, Resources, Software, Writing – review & editing. PG: Methodology, Resources, Supervision, Writing – review & editing. MA: Methodology, Resources, Supervision, Writing – review & editing. KS: Investigation, Methodology, Writing – review & editing. KR: Investigation, Methodology, Writing – review & editing. SE: Investigation, Project administration, Writing – review & editing. IN: Investigation, Methodology, Resources, Supervision, Writing – review & editing. AG: Funding acquisition, Project administration, Resources, Writing – review & editing.

## Footnotes

^1^ www.qiagen.com/HB-1792

^2^ https://www.bioinformatics.com.cn/plot_multi_types_2d_pie_by_tastypie_128_en

## Supplementary material

SUPPLEMENTARY FILE 1 Core microbes across different ontogeny and their prevalence.

SUPPLEMENTARY FILE 2 PICRUSt2 analysis of the metagenomics data of *T. blochii* across different ontogeny.

SUPPLEMENTARY FILE 3 Taxonomic landscape in the microbiota profiles of *T. blochii* across different studied groups.

SUPPLEMENTARY FILE 4 Shared bacterial genera across different ontogeny and their prevalence.

SUPPLEMENTARY FILE 5 Shared bacterial genera across the studied sources of fish microbiotaand their prevalence.

SUPPLEMENTARY FIGURE 1 Taxonomic landscape in the microbiota profiles across different ontogenetic stages of *T. blochii* **(A)** At class level; **(B)** At order level; **(C)** At family level. DPH, Days post-hatching.

SUPPLEMENTARY FIGURE 2 Principal Coordinate Analysis based on the weighted UniFrac distance of the ASV abundance profiles.

SUPPLEMENTARY FIGURE 3 Microbial biomarkers discriminating between the egg microbiota and different ontogenetic stages **(A)** Microbial biomarkers discriminating between the egg microbiota and ≤10 DPH whole microbiota profiles; **(B)** Microbial biomarkers discriminating between the egg microbiota and >10 DPH whole microbiota profiles; **(C)** Microbial biomarkers discriminating between the egg microbiota and gut microbiota profiles.

SUPPLEMENTARY FIGURE 4 Taxonomic landscape in the microbiota profiles of the microalgae, rotifer, artemia, formulated feed, and rearing water **(A)** At class level; **(B)** At order level; **(C)** At family level. DPH, Days post-hatching.
