# Supplementary material for: Mechanistic insights into the early life stage microbiota of silver pompano (Trachinotus blochii)
Source: Front Microbiol. 2024 Apr 17;15:1356828. doi: 10.3389/fmicb.2024.1356828 (PMC11061439; doi:10.3389/fmicb.2024.1356828)
Supplement: SUPPLEMENTARY FILE 1 — Core microbes across different ontogeny and their prevalence. [file Data_Sheet_1.docx]

**Supplementary tables (ST)**

Supplementary table 1: Morphometrics of animals and rearing water quality conditions at each sampling time point in the present study

| Life stage (DPH) | Length (mm) | Water Temperature | TAN level (mg/L) | Water salinity |
| --- | --- | --- | --- | --- |
| 1 | 2.29 ± 0.58 | 28 ± 0.05°C | 0.005 ± 0.01 | 34 ± 1 ppt |
| 3 | 2.73 ± 0.07 |  |  |  |
| 5 | 3.07 ± 0.15 |  |  |  |
| 7 | 3.48 ± 0.24 |  |  |  |
| 10 | 4.68 ± 0.36 |  |  |  |
| 12 | 5.36 ± 0.46 |  |  |  |
| 20 | 11.3 ± 0.78 |  |  |  |
| 25 | 15.5 ± 0.75 |  |  |  |
| 31 | 22.35 ± 0.5 |  |  |  |
| 75 | 82. 3 ± 0.3 |  |  |  |

Abbreviations: DPH: Days post hatching; TAN: Total ammonia‑nitrogen; DO: Dissolved oxygen

Supplementary table 2**:** PERMANOVA results based on Bray-Curtis similarity matrix for difference in microbiota compositions across the studied groups

|  | EM | ≤10 DPH WM | >10 DPH WM | GM |
| --- | --- | --- | --- | --- |
| EM |  | 3.634 | 1.477 | 2.506 |
| ≤ 10 DPH WM | 0.0013 |  | 5.89 | 3.469 |
| >10 DPH WM | 0.0316 | 0.0001 |  | 3.083 |
| GM | 0.0114 | 0.0001 | 0.0004 |  |

Abbreviations: EM: Egg microbiota; DPH: Days post hatching; GM: Gut microbiota
