# Supplementary figures and images for: Mechanistic insights into the early life stage microbiota of silver pompano (Trachinotus blochii)
Source: Front Microbiol. 2024 Apr 17;15:1356828. doi: 10.3389/fmicb.2024.1356828 (PMC11061439; doi:10.3389/fmicb.2024.1356828)

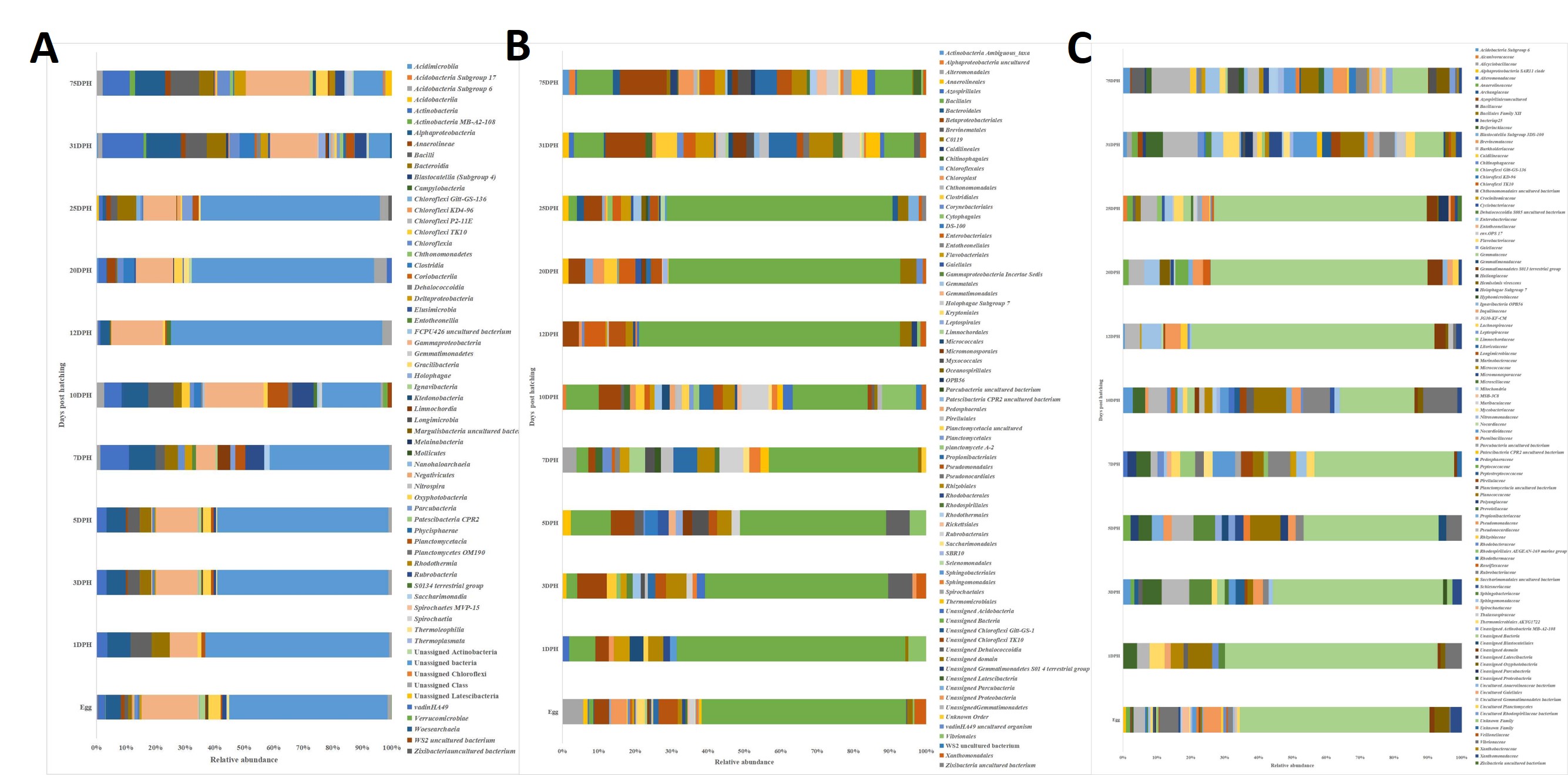

Supplement: SUPPLEMENTARY FIGURE 1 — Taxonomic landscape in the microbiota profiles across different ontogenetic stages of T. blochii (A) At class level; (B) At order level; (C) At family level. DPH, Days post-hatching. [file Image_1.JPEG]

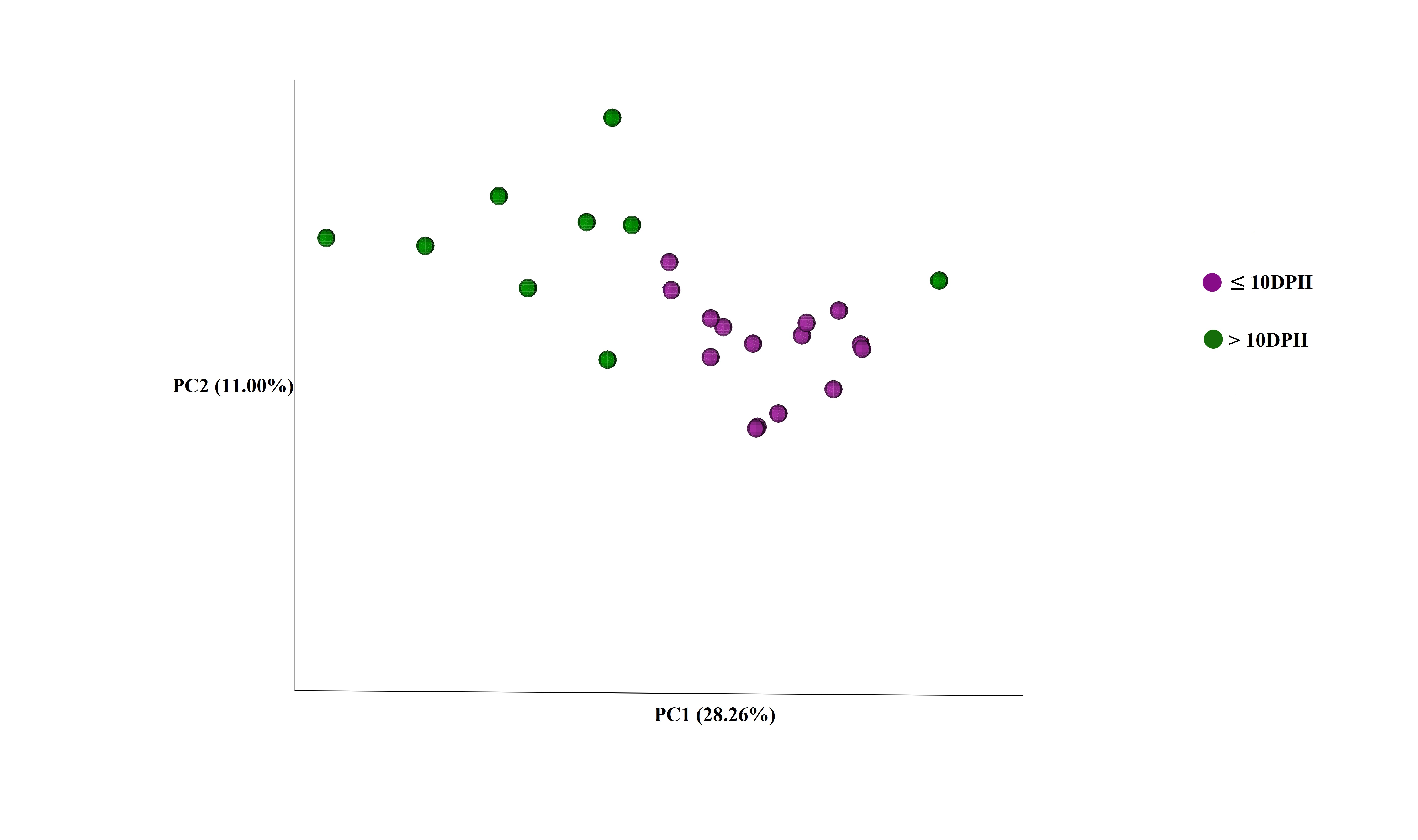

Supplement: SUPPLEMENTARY FIGURE 2 — Principal Coordinate Analysis based on the weighted UniFrac distance of the ASV abundance profiles. [file Image_2.JPEG]

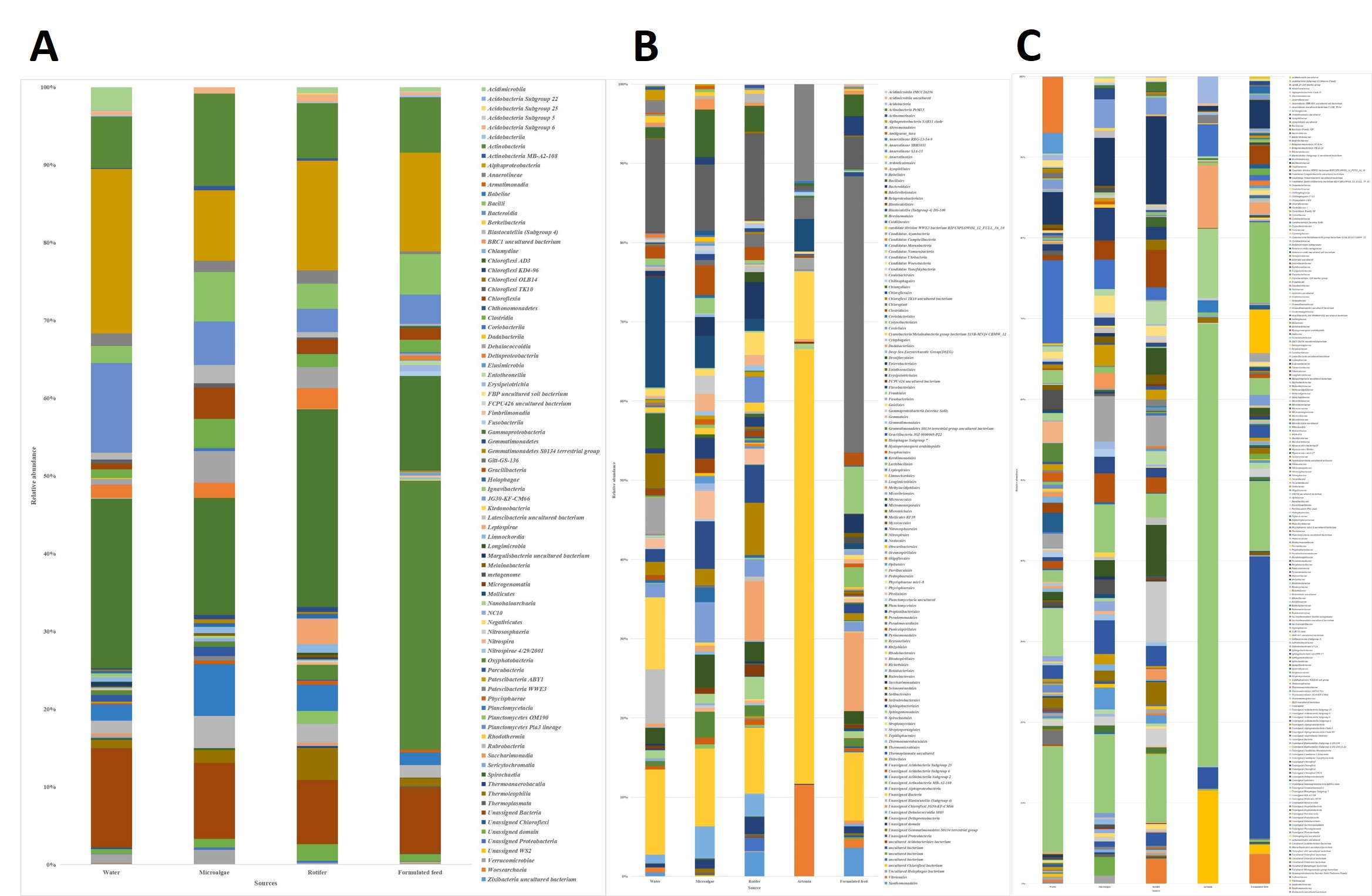

Supplement: SUPPLEMENTARY FIGURE 3 — Microbial biomarkers discriminating between the egg microbiota and different ontogenetic stages (A) Microbial biomarkers discriminating between the egg microbiota and ≤10 DPH whole microbiota profiles; (B) Microbial biomarkers discriminating between the egg microbiota and >10 DPH whole microbiota profiles; (C) Microbial biomarkers discriminating between the egg microbiota and gut microbiota profiles. [file Image_3.JPEG]

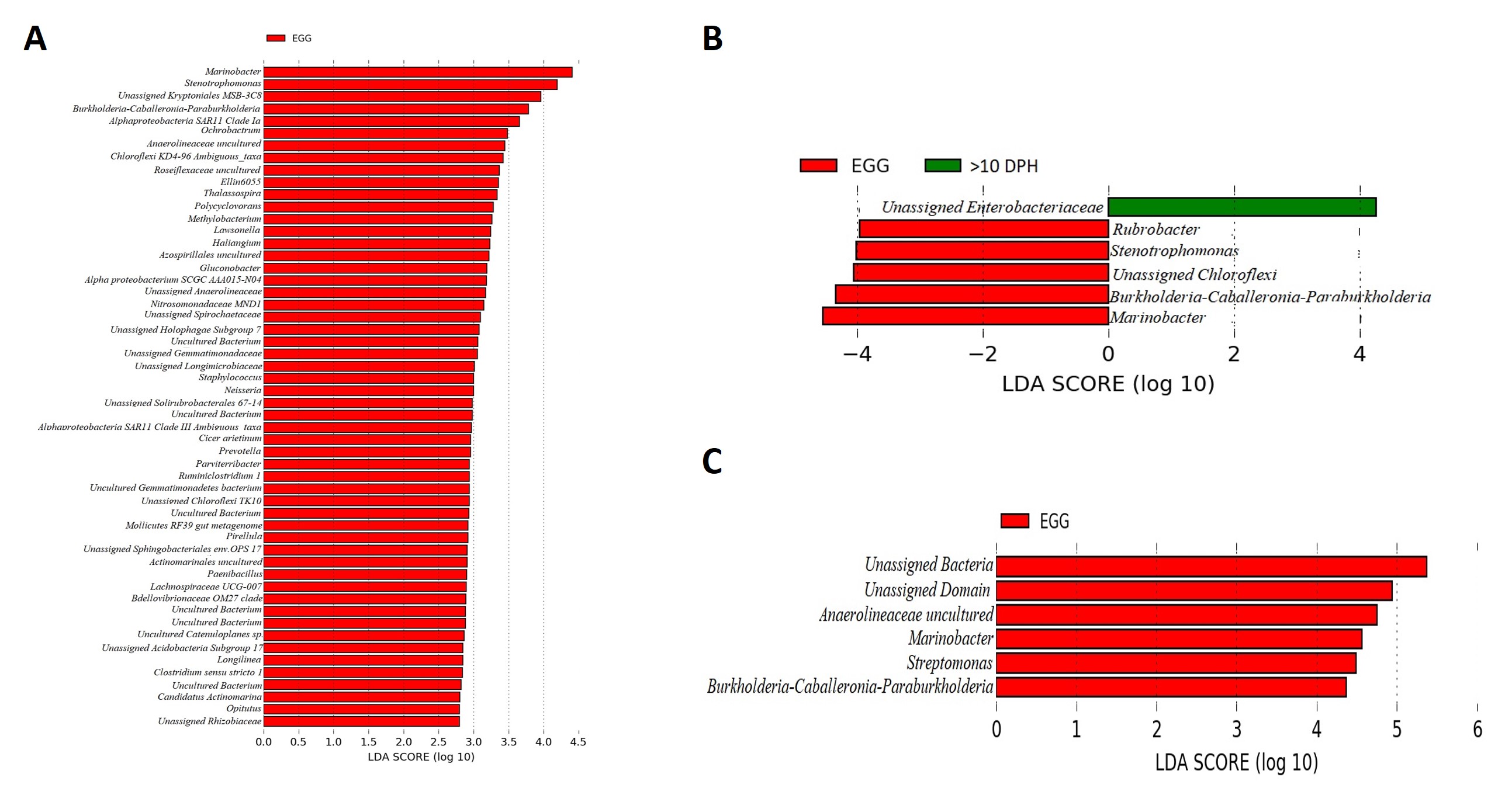

Supplement: SUPPLEMENTARY FIGURE 4 — Taxonomic landscape in the microbiota profiles of the microalgae, rotifer, artemia, formulated feed, and rearing water (A) At class level; (B) At order level; (C) At family level. DPH, Days post-hatching. [file Image_4.JPEG]
